# Vitrimeric Behavior Revealed by Fast Scanning Calorimetry in Branched Polyglycerol Networks Cross-Linked by Reversible Enamine Bonds

**DOI:** 10.1021/acs.macromol.5c01560

**Published:** 2025-09-03

**Authors:** Vasiliki Maria Stavropoulou, Marta Aldecoa-Ortueta, Ester Verde-Sesto, Valerio Di Lisio, Anabel Lam, José A. Pomposo, Angel Alegría, Daniele Cangialosi, Fabienne Barroso-Bujans

**Affiliations:** † Materials Physics Center (CFM-MPC), CSIC-UPV/EHU, Paseo Manuel Lardizábal 5, 20018 Donostia−San Sebastián, Spain; ‡ PMAS, Faculty of Chemistry, University of the Basque Country (UPV/EHU), Paseo Manuel Lardizábal 3, 20018 Donostia−San Sebastián, Spain; § IKERBASQUE—Basque Foundation for Science, Plaza Euskadi 5, 48009 Bilbao, Spain; ∥ 226245 Donostia International Physics Center (DIPC), Paseo Manuel Lardizábal 4, 20018 Donostia−San Sebastián, Spain; ⊥ Zeolites Engineering Laboratory, Institute of Materials Science and Technology (IMRE), 113031 University of Havana, 10400 La Habana, Cuba

## Abstract

Dynamic covalent bonds formed by enamine have played a crucial role in the development of vitrimers by enabling their rearrangement and reprocessability. In this study, polymer networks obtained by cross-linking a β-ketoester-functionalized branched polyglycerol (PG-βkest) with three different diamines, i.e. diaminopropane (DAP), 2,2′-(ethylenedioxy)­bis­(ethylamine) (EDO) and Jeffamine D230 (Jeff) were generated through enamine bond formation enabling the formation of materials with tunable glass transition temperatures (*T*_g_). A single, though broad, specific heat step was detected in all cases by conventional differential scanning calorimetry (DSC). However, by judiciously varying thermal protocols using fast scanning calorimetry (FSC), which permits heating/cooling rates as large as 1000 K s^–1^, we were able to separate and identify two distinct thermal events in the network composed by Jeff, used as a vitrimeric network model. Using Kissinger analysis, we conveyed information about the apparent activation energies of the two thermal events. In such a way, we were able to provide compelling evidence that the high-temperature event is due to a vitrimeric transformation (*T*_v_), while the low-temperature event exhibits all the features of a conventional glass transition.

## Graphical abstract

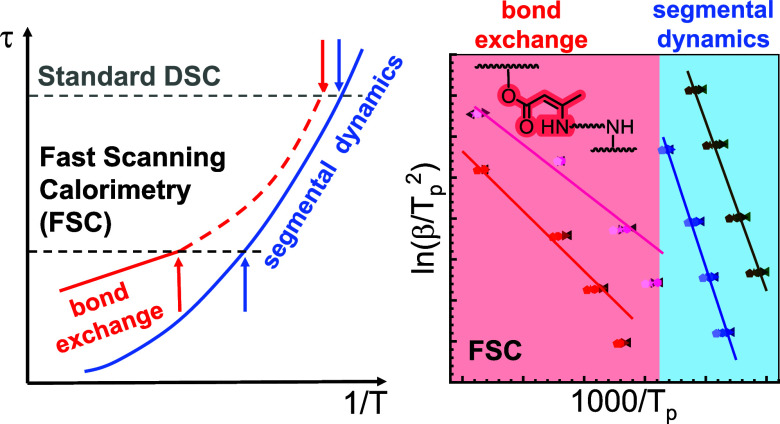

## Introduction

Dynamic covalent polymer networks, also known as covalent adaptable networks (CANs), have emerged as an advanced form of classical polymer networks. They possess unique features such as reprocessability, self-healing, and reusability.^1^ CANs are based on dynamic covalent bonds (DCBs) which allow the network topology to reorganize. Under appropriate conditions, CANs can flow, be remodeled and/or (self-)­repair.^2^ DCBs in CANs can be dissociative or associative, depending on whether they are disrupted before the exchange event or remain intact, respectively.^3,4^ In particular, CANs possessing associative DCBs and showing an Arrhenius temperature dependence of viscosity approaching the glass transition temperature (*T*_g_) are called vitrimers.^5^ A relevant parameter of these systems is the topology freezing or vitrimer transition temperature (*T*_v_), which Leibler and co-workers defined as the temperature at which the network freezes due to the inability of DCBs to undergo exchange reactions.^6^ Increasing the temperature just above *T*_v_ causes vitrimers to undergo a topological transition from viscoelastic solid to viscoelastic liquid behavior.

Reliably determining *T*_v_ remains an unsolved problem in this emerging field. An extrapolation of the vitrimer viscosity to a value of 10^12^ Pa s is taken as the original criterion to estimate the so-called “apparent” *T*_v_, even if large extrapolations prone to large errors are often involved.^7,8^ Other attempts to obtain *T*_v_ have relied on the use of stress–relaxation measurements combined with nonisothermal and isothermal creep experiments,^9^ dilatometry,^10^ thermomechanical analysis,^11^ aggregation-induced-emission (AIE) luminogen fluorescence^12^ and X-ray scattering experiments supported by DSC measurements.^13^

Methods that provide first-order thermodynamic properties, such as dilatometry for specific volume and DSC for enthalpy, rely on the fact that the vitrimeric transition, in a manner analogous to the glass transition, activates translational and rotational degrees of freedom.^14^ This is expected to result in a stepwise increase in the thermodynamic coefficients, that is, the coefficient of thermal expansion and the specific heat capacity. Despite this, the simultaneous determination of *T*_g_ and *T*_v_ has hitherto remained vastly elusive with only a few exceptions.^10,15^ This is likely because at the rates of K min^–1^ explored by standard dilatometry and DSC, *T*_g_ and *T*_v_ are often too close and, therefore, their separation may be challenging. Furthermore, the step-like variation in thermodynamic coefficients underlying the vitrimeric transformation can span a broad temperature range, which complicates its detection. These experimental flaws can be overcome by expanding the range of the heating/cooling rate in the experiment and by applying thermal protocols that aim to magnify the thermal events under investigation. As with *T*_g_, it is expected that *T*_v_ depends on several experimental parameters in addition to the specific chemistry of the material.^16^ Moreover, *T*_v_ can be above, below or near *T*_g_. In fact, there is no general agreement on a protocol or experimental setup to assess *T*_v_.

With regards to the separation between glass and vitrimeric transitions specifically, the latter generally exhibits a lower apparent activation energy than the segmental relaxation associated with the glass transition (see Figure S1). Therefore, reducing the experimental time scale can be an effective way of identifying the thermal events involved in vitrimers. FSC enables heating and cooling scans as large as several thousand K s^–1^,^17^ which greatly reduces the time scale of probed molecular motion compared to conventional DSC.

Enamine DCBs^18^—renamed as “vinylogous urethane” bonds by Du Prez and co-workers^19^—has become one of the canonical reversible bonds used in the development of vitrimers made from synthetic^20,21^ and biobased materials.^22,23^ Branched polyglycerol (PG),^24^ which has multiple hydroxyl groups and a branched structure, can be easily decorated with β-ketoester functional groups through a transesterification reaction with *tert*-butyl acetoacetate (TBAA). In this work, we have synthesized networks cross-linked through enamine DCBs based on β-ketoester-decorated branched polyglycerol (PG-βkest) and different diamines, i.e. DAP, EDO and Jeff.

Furthermore, we have developed an experimental protocol to assess *T*_v_ using FSC of the synthesized cross-linked networks with Jeff. We have found that using DSC, these systems have *T*_v_ very close to *T*_g_. However, the thermal protocols permitted by FSC allowed us to unambiguously resolve both thermal phenomena and to determine the activation energy of the vitrimeric transformation.

## Experimental Section

### Materials

(±)-Glycidol (Gly) (96%), *tert*-butyl acetoacetate (TBAA, 98%) and 1,3-diaminopropane (DAP, >99%), 2,2′-(ethylenedioxy)­bis­(ethylamine) (EDO), *N*,*N*-dimethylformamide (DMF) and CaH_2_ were purchased from Sigma-Aldrich. B­(C_6_F_5_)_3_ (>98.0%) was obtained from TCI Europe and purified by sublimation under reduced pressure at 90 °C. Jeffamine D230 (Jeff) was purchased from Huntsman. Toluene, tetrahydrofuran (THF), ethanol (EtOH), diethyl ether (Et_2_O) were purchased from Scharlab and methanol (MeOH) from Fischer Scientific. Deuterated chloroform (CDCl_3_) and deuterated water (D_2_O) were obtained from Euroisotop. Gly and toluene were distilled from CaH_2_ under reduced pressure. They were stored under inert atmosphere and transferred either in glovebox or in a vacuum line. The rest of reagents and solvents were used as received.

### Synthesis of PG

The synthesis of PG was performed in a three-necked flask equipped with a jacket and a magnetic stirrer under argon atmosphere. A flask with 6 mL of Gly (6.66 g, 90 mmol) and 20 mL of toluene was cooled to 0 °C, then 59 mg of B­(C_6_F_5_)_3_ (0.12 mmol) dissolved in 4 mL of toluene and 60 μL of water were added after stabilizing the temperature. The reaction was stirred for 22 h obtaining 99 mol % monomer conversion as determined by ^1^H NMR. The polymer precipitated during the reaction as a viscous material generating two phases, the solvent and the precipitate, as expected from our previous study.^25^ The polymer was separated from the solvent by decantation. Afterward, the resulting polymer was dissolved in MeOH (10 mL) and purified by precipitation in Et_2_O (100 mL). Then, the polymer was again dissolved in MeOH (10 mL) and passed through basic alumina. The solvent was removed under vacuum in the rotary evaporator and finally, the isolated product was dried at 80 °C for 18 h in a vacuum oven. The product was obtained as a transparent viscous material (3.3 g, yield 60 wt %). *M*_n_ = 3 kg/mol (*D̵* = 2.0) was determined by gel permeation chromatography (GPC) and a degree of branching of 0.39 by inverse-gated ^13^C NMR in D_2_O following our previous works.^25,26^

### Synthesis of PG-βkest

PG (830 mg, 11 mmol of OH) and a large excess of TBAA (36 mL, 217 mmol) were added to a Schlenk flask of 250 mL. The reaction mixture was stirred at 120 °C for 22 h. Then, the reaction mixture was cooled down to room temperature and transferred to a round-bottom flask of 100 mL. The final product was purified by distillation at 130 °C and 50 mbar for 1 h. This process was used to remove the TBAA excess and the formed *tert*-butanol. The product was then washed with Et_2_O. PG-βkest was obtained as a viscous yellow oil material (1.3 g, 74 wt % yield).

### Synthesis of Cross-Linked Networks with Diamines

As an example of a network prepared with DAP/βkest (feed) molar ratio = 1.2, PG-βkest (83 mg) was first dissolved in 9.6 mL of THF in a vial to generate 0.05 mol­(βkest)/L solution. Separately, in another vial, DAP (24 μL) was added to 288 μL of THF to generate a 1 mol/L of DAP solution. Then, the diamine solution was added to that of the polymer. The mixture was transferred into a Teflon Petri dish of 5 cm diameter, and the solvent was evaporated at room temperature overnight. The resulting film was cured at 85 °C for 1 h under vacuum conditions in a vacuum oven. See Table S1 for the amounts of reagents used for all samples prepared with DAP, and Tables S2 and S3 for samples prepared with Jeff and EDO. All the networks were prepared using the same batch of PG-βkest. The excess of Jeff was successfully removed using Soxhlet extraction with EtOH. The excess of DAP and EDO was removed by evaporation following a thorough evaluation of the thermal protocol to be used. The networks cross-linked with DAP and EDO were heated at 150 °C for 15 min to 1 h (depending on the sample) in a vacuum oven.

### Characterization Techniques

^1^H and inverse gated ^13^C NMR data were acquired on a Bruker Avance Neo 500 at 25 °C, employing D_2_O for PG and CDCl_3_ for PG-βkest. The degree of branching (DB) was calculated from $DB=\frac{2D}{2D+L_{1,3}+L_{1,4}}$ , where, *D*, *L*_1,3_ and *L*_1,4_ are the relative abundance of dendritic and linear structures, respectively.^27^

GPC data were acquired on a Nexera instrument from Shimadzu using refractive index detector (RID-20A, Shimadzu) and MALS detector (λ = 663.89 nm, miniDawn, Wyatt) at a temperature of 40 °C. Separation was performed at 50 °C by using a CTO 40C column oven and Polargel-M Guard 50 × 7.5 mm and Polargel-M 300 × 7.5 mm, 8 μm, GPC columns. HPLC grade DMF containing 0.1% of LiBr with a flow of 1.0 mL/min was used as a mobile phase. The absolute molecular weight of PG was determined using a d*n*/d*c* value^28^ of 0.054 mL/g and Astra 8.1 software from Wyatt Technology.

The determination of the amount of carbon, hydrogen and nitrogen of the networks was performed on a EuroEA 3000 elemental analyzer.

Fourier Transform Infrared Spectroscopy (FTIR) spectra were recorded at room temperature in the 600–4000 cm^–1^ spectral region on a JASCO 3600 FTIR spectrometer equipped with an ATR accessory. Each sample was analyzed with a resolution of 4 cm^–1^ and an average of 200 scans. The baseline of the spectra was not corrected and the spectrum was not smoothed.

Thermogravimetric analysis (TGA) data were recorded on a TA Instruments TGA Q500, under a nitrogen atmosphere (constant flow of 60 mL/min). Samples were heated from 25 to 600 °C with a heating ramp of 10 °C/min.

Standard DSC measurements were performed on ∼5 mg samples using a Q2000 TA Instrument. PG and PG-βkest were measured in aluminum pans without a lid, after it was confirmed that the type of pan significantly affects the reproducibility of the PG data.^26^ The cross-linked networks were measured in sealed aluminum pans for solid samples. The sample was first cooled from room temperature to −100 °C (or −50 °C for the networks) and then heated to 150 °C at 10 °C/min (first heating run). Then, samples were cooled back to −100 °C (or −50 °C for the networks) at 10 °C/min and heated to 150 °C at 10 °C/min (second heating run). A third cooling and heating cycle was then performed to verify reproducibility. A helium flow rate of 25 mL/min was used throughout. Glass transition temperatures (*T*_g_) were determined from the maximum of the first derivative of the heat flow rate in the second heating run.

FSC measurements were carried out employing a Mettler Toledo Flash Differential Scanning Calorimeter (Flash DSC 1) complemented with an Uber TC100 intracooler, allowing to operate in a temperature range between 173 and 723 K. Dry nitrogen was pumped into the sample chamber at a flow rate of 20 mL/min. All samples were prepared by positioning a mass of ∼100 ng directly onto a Mettler Toledo UFS 1 chip. To minimize thermal gradients within the sample, special care was taken to keep the sample height below 10 μm. The absence of significant temperature gradient was verified depositing a little piece of indium on top of the sample. This served also for temperature calibration together with indium deposited on the reference area. All experiments began with a cooling scan from high temperature. The latter was judiciously chosen to guarantee that an equilibrium system is attained and to avoid polymer degradation. With the 2-fold aim of magnifying thermal events in the vitrimers and treating data via the Kissinger analysis (see Results and Discussion section), the cooling rate varied between 1000 and 10^–3^ K s^–1^ and heating rate between 50 and 1000 K s^–1^. The absence of thermal degradation was verified by comparing reference scans at the beginning and at the end of each set of experiments. It is noteworthy that FSC generally enables success to avoid thermal degradation. The reason stands in the fact that the activation energy of the thermal degradation process is typically much smaller than that of both vitrimeric and glass transition. Details on the interplay between degradation and thermal events with large activation energies are reported in ref 29.

To gain insights into active calorimetric molecular relaxation processes, we applied the so-called step-response protocols.^30,31^ This is based on measuring the heat flow after subjecting the sample to a small temperature down-jump followed by an isotherm. In our study the chosen temperature step was 2 K, a perturbation small enough to guarantee the linearity of the measurement.^32^ Since our protocol was applied in both standard DSC and FSC, the cooling rate for the temperature step and the duration of the isotherm were optimized to minimize the signal-to-noise ratio and the thermal lag, and to explore the widest possible frequency range. The ratio of Fourier transform of the instantaneous heat flow to the instantaneous cooling rate gives a quantity proportional to the frequency (f) dependent complex specific heat (eq 1), where ω is the angular frequency (ω = 2πf).

1 $$C_{p}^{*}\left(\omega \right)=C_{p}^{\prime }\left(\omega \right)+iC_{p}^{\prime \prime }\left(\omega \right)$$

The combination of standard DSC and FSC allowed us to access a frequency range between 10^–3^ and 50 Hz.

Dynamic mechanical (DMA) experiments were performed using an ARES-LS2 torsional rheometer (TA Instruments) with a parallel plate geometry (8 mm diameter). Experiments were performed by using samples of about 0.1 mm thickness with a 0.1% strain at 1 rad/s frequency and recording the complex shear modulus during temperature ramps at a rate of 1 K/min.

## Results and Discussion

### Functionalization of Branched Polyglycidol

PG was functionalized with beta-ketoester groups (PG-βkest) by a transesterification reaction with *tert*-butyl acetoacetate (TBAA) (Scheme 1a). ^1^H, ^13^C, COSY, HSQC and DEPT 135° NMR and FTIR analysis confirmed the formation of targeted structures (Figures 1 and S2–S4). ^1^H NMR data exhibited the formation of the enol form of the βkest (signals identified as a′, b′ and c′ in Figure 1) indicating the occurrence of keto–enol tautomerism in solution (CDCl_3_), as previously observed in other polymeric systems.^33^ Comparison of the peak integrals of –CH_3_ in the keto (δ = 2.28 ppm) and enol forms (δ = 1.98 ppm) indicated that 89% existed in the keto form, which is the functionality that will react with amines to form enamines. The degree of functionalization was determined by comparing the proton integrals of both keto and enol forms (a + a′) relative to the CH and CH_2_ protons from the polymer backbone. The results indicated that 83% of the hydroxyl groups of the polymer chain were reacted.

**Scheme 1:**
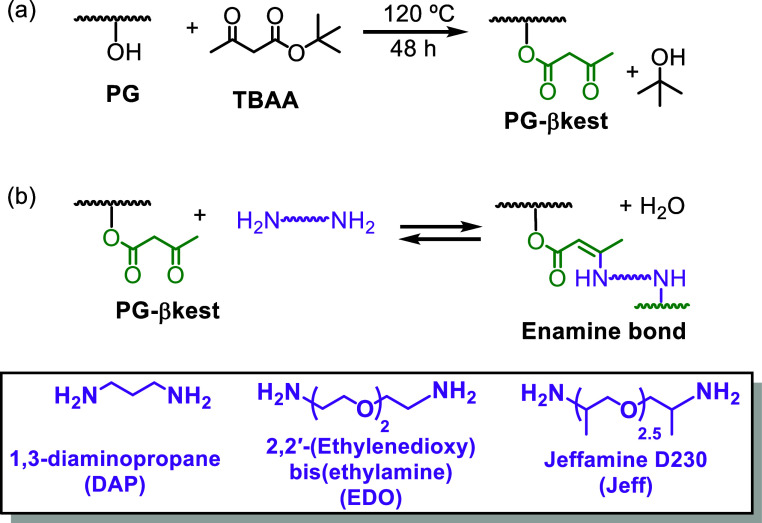
(a) Functionalization of PG with TBAA. (b) Formation of Enamine Bonds by Reaction of PG-βkest with a Diamine. In the Box at the Bottom: Diamines Used in Present Study

**Figure 1:**
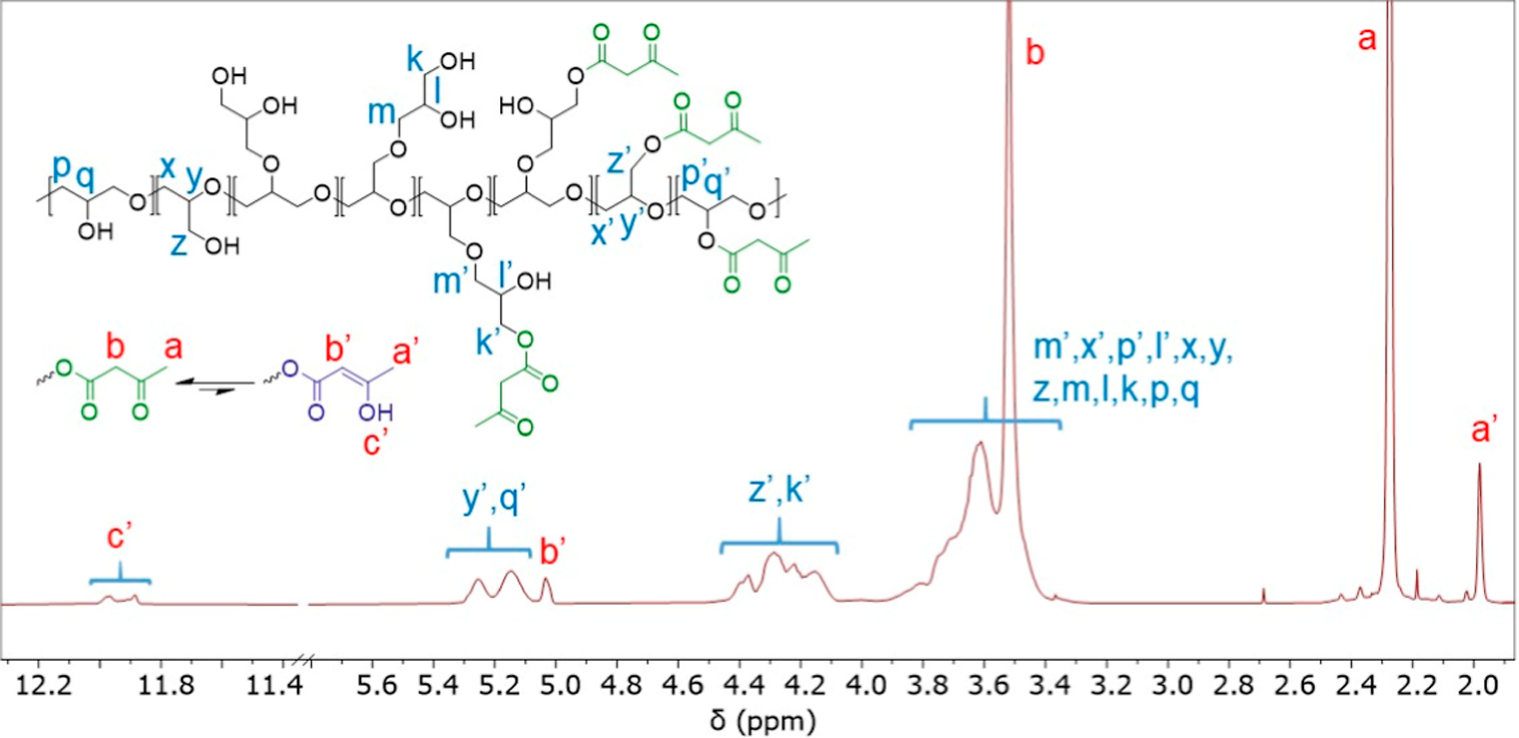
^1^H NMR (CDCl_3_) spectrum of PG-βkest, showing the formation of tautomeric structures.

### Synthesis of Cross-Linked Networks with Diamines

Polymer networks were obtained by reaction of PG-βkest with 1,3-diaminopropane (DAP), 2,2′-(ethylenedioxy)­bis­(ethylamine) (EDO), or Jeffamine D230 (Jeff) (Scheme 1b). After mixing the polymer with the diamines at room temperature (25 °C) in Teflon Petri dishes for 24 h, homogeneous films were observed. Different mole equivalents of diamine were used to cross-link 1 mol equivalent of βkest motifs through enamine bonds. Excess of diamine was removed by either evaporation or Soxhlet extraction. As a result, films with a thickness of approximately 100 μm were obtained, displaying distinct textural characteristics contingent solely on the nature of the amine employed in the synthesis (Figure 2a). The composition of the polymer networks was calculated by employing the amount of nitrogen in the sample as determined by elemental analysis (eqs S1–S7). Figure 2b shows the moles of amine per mol of βkest obtained in the sample as a function of the moles of amine per mol of βkest used to prepare the networks (feed). Sample values below feed values of 1 exhibit a growing trend with a slope near 1 indicating that all the diamine used in the feed is retained in the polymer network through covalent bonds. However, at feed values above 1, the amount of amine in the sample does not follow this trend and remains in the range of 0.9–1.1 indicating that the excess of diamine has been removed during purification.

**Figure 2:**
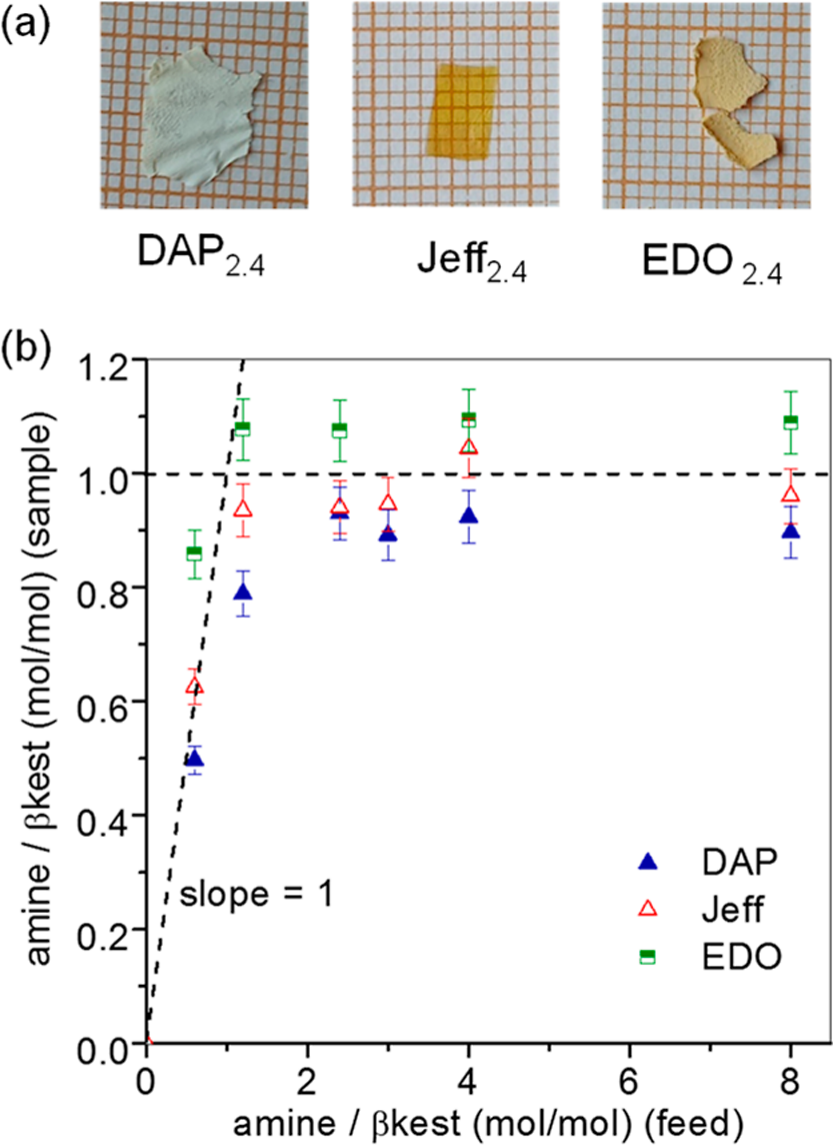
(a) Photographs of films over millimeter graph paper (samples obtained with an amine/βkest (feed) molar ratio of 2.4). (b) Composition of polymer networks cross-linked with DAP, EDO and Jeff.

TGA data of PG-βkest and cross-linked networks exhibited important changes in their thermal stability compared to nonfunctionalized PG precursor (Figures 3 and S5). PG is thermally stable up to 300 °C (onset temperature) whereas its βkest derivative starts to decompose at 170 °C due to the incorporation of thermally unstable carbonyl groups into the polymer structure. A weight loss of 51% is in agreement with the calculated weight loss of βkest group relative to the total mass of the polymer. TGA data of cross-linked networks showed that upon the formation of enamine groups, the thermal stability slightly increased. The network obtained with an amount of amine below the stoichiometric one (amine/βkest (feed) molar ratio = 0.6) starts to decompose at a similar temperature than that of the PG-βkest precursor. However, the networks cross-linked with molar equivalents of amine per mole of βkest (feed) ≥ 1.2 exhibited higher thermal stability than the previous one and an identical decomposition profile, indicating a similar number of cross-linking points.

**Figure 3:**
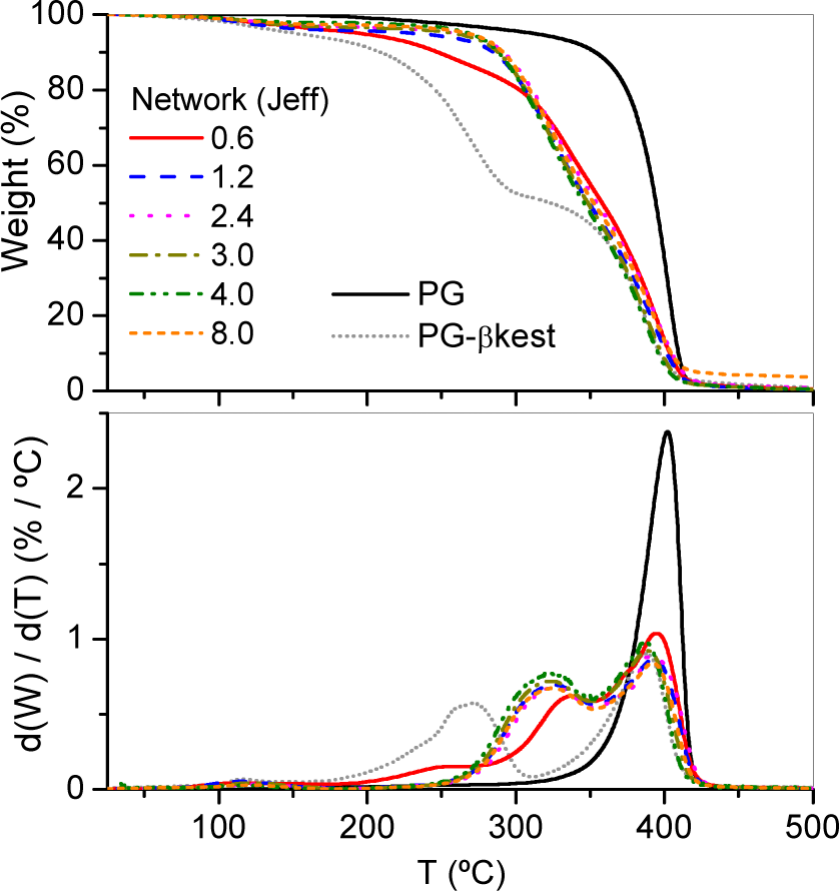
TGA data recorded at 10 °C/min under nitrogen atmosphere of PG, PG-βkest and cross-linked networks obtained by reaction of PG-βkest with Jeff using different amine/βkest molar ratios in the feed (from 0.6 to 8.0). (top) Weight loss. (bottom) First derivative of weight with respect to temperature.

FTIR data of the cross-linked networks revealed the gradual disappearance of the β-ketoester peaks at 1739 (ν_C=O ester_) and 1714 cm^–1^ (ν_C=O ketone_) with the increasing amount of the diamine and the appearance of two bands centered at 1649 (ν_C=O ester_) and 1597 cm^–1^ (ν_C=C_) in all the systems investigated (Figures 4, 5 and S6), in agreement with the formation of enamine bonds.^34^ Other features that characterize the cross-linked networks are the disappearance of the NH_2_ wagging vibration of DAP (849 cm^–1^) and Jeff (830 cm^–1^) upon reaction, which is typical of primary amines, and the appearance of a CH out-of-plane deformation band of CH=C bonds at 781 cm^–1^. The disappearance of the NH_2_ internal deformation band detected at 1599, 1594, and 1598 cm^–1^ for DAP, Jeff and EDO, respectively, cannot be observed in the cross-linked networks due to the superposition of an absorption ν_C=C_ band at 1597 cm^–1^. The NH deformation band of the formed secondary amine is too weak and cannot be detected readily.

**Figure 4:**
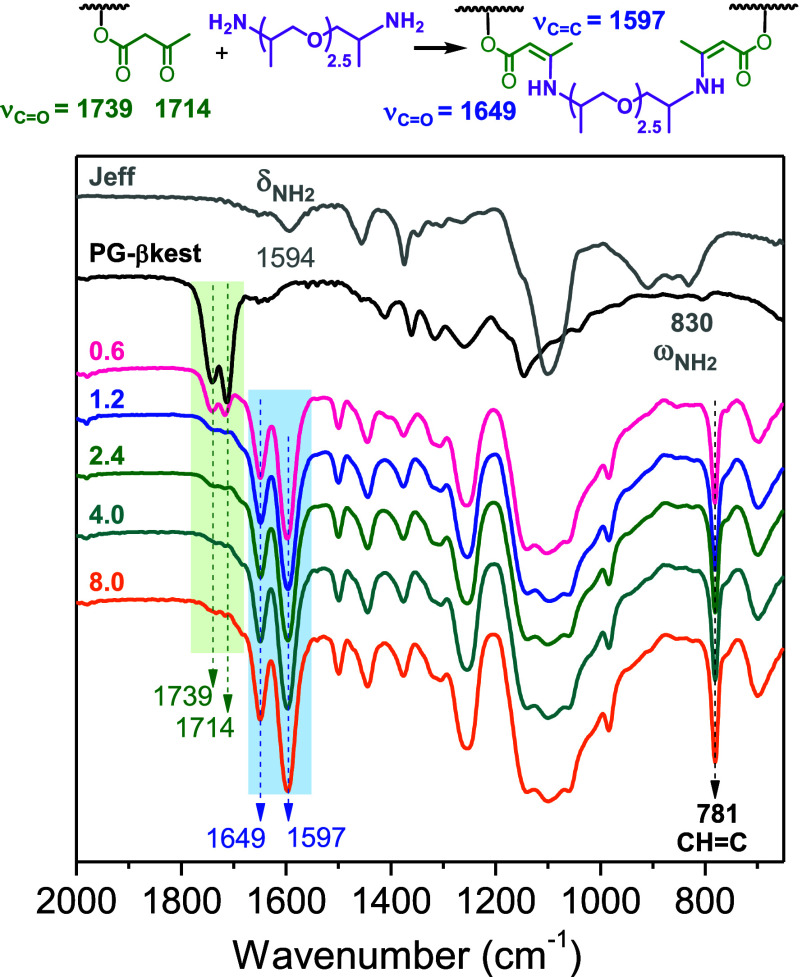
FTIR spectra of the cross-linked networks formed by PG-βkest and Jeff obtained with different amine/βkest molar ratios in the feed (from 0.6 to 8.0).

**Figure 5:**
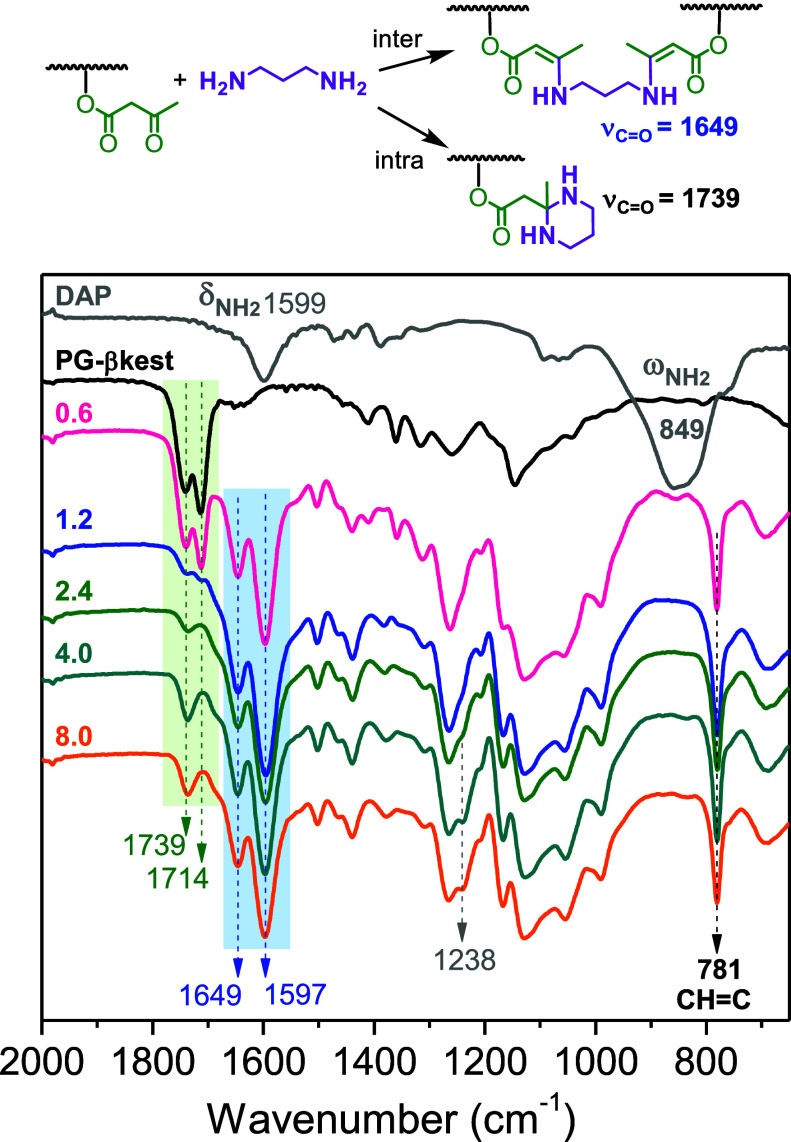
FTIR spectra of the cross-linked networks formed by PG-βkest and DAP obtained with different amine/βkest molar ratios in the feed (from 0.6 to 8.0).

In the case of the network obtained with DAP, the appearance of a band at 1739 cm^–1^ (ν_C=O ester_) and 1238 cm^–1^ (skeletal vibrations involving ν_(NH)_2_C–CH_3__ and ν_(NH)_2_C–CH_2__) was observed for a high excess of DAP in the feed, with amine/βkest molar ratios ≥2.4 (Figure 5). This result suggests that upon curing at 85 °C and further heating at 150 °C to remove excess DAP (see Figure S7), the formation of 2-methyl hexahydropyrimidine moieties is favored through an intramolecular cyclization reaction of the diamine. The results also indicate that this intramolecular reaction is more likely in an excess of DAP, where the diamine is expected to be largely anchored by only one amine group, enabling the second amine group to attack the quaternary carbon and to form an aminal group. The reaction of 1,3-diamines in poly­(vinyl amine) with acetone to form aminal groups has been reported,^35^ supporting the present findings. The aminal formation was not observed for the cross-linked networks formed by Jeff and EDO (Figures 4 and S6) due to the unlikely formation of less thermodynamically stable aminal groups compared to enamine groups. Discussion of thermodynamic aspects of these reactions using DFT calculations is given in the Supporting Information and Figure S13.

### Glass Transition Temperature

The characterization of the *T*_g_ of the cross-linked networks obtained with different amounts of diamine is shown in Figure 6a. For a given amount of amine in the feed, a significant increase in *T*_g_ can be observed when moving from Jeff to EDO to DAP. This is expected considering the flexibility of these amines. It is also noteworthy that, while the glass transition range for Jeff and EDO is relatively narrow (similar to that of the precursor for Jeff and slightly broader for EDO), it is remarkably broader for DAP. This finding indicates that the latter system exhibits an anomalous, heterogeneous glass-to-rubber transformation, likely due to the formation of non-cross-linked aminal structures, as described above.

**Figure 6:**
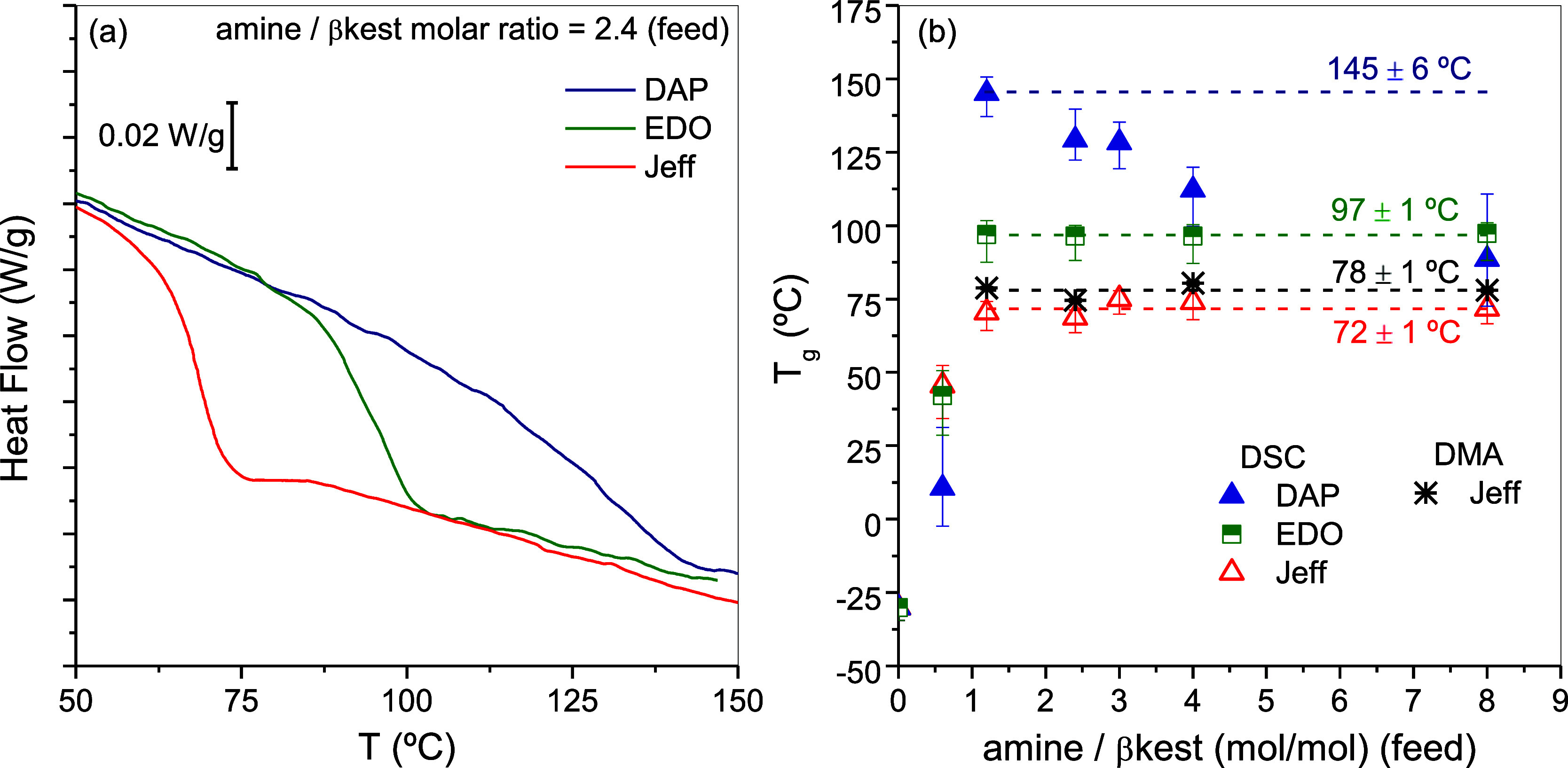
(a) DSC traces obtained in three cross-linked networks obtained using different amines but maintaining a constant ratio amine/βkest (feed) = 2 (b) *T*_g_ of cross-linked networks as a function of amine/βkest (feed) molar ratios determined by DSC and DMA. Error bars represent the *T*_g_ width. Dashed lines for Jeff and EDO are linear fittings at amine/βkest ≥ 1.2 (feed) with slopes fixed to zero. Dashed line for DAP shows the maximum *T*_g_ obtained. Asterisks correspond to values obtained for the Jeff networks by DMA at 1 Hz.

Figure 6b summarizes the dependence of *T*_g_ on the amount of amine in the feed for networks cross-linked with Jeff, EDO and DAP (see also Figure S8). Those cross-linked with Jeff and EDO showed the expected increase in *T*_g_ with increasing amount of amine in the feed to reach a plateau where a further increase in amine content does not result in further cross-links; the excess of amine being removed to reach final amine/βkest molar ratios near 1 (Figure 2b). In contrast, the networks cross-linked with DAP showed an initial rapid increase in *T*_g_ with increasing amounts of amine up to values as high as 145 °C, followed by a decrease in *T*_g_. The favored intramolecular reaction at amine/βkest (feed) molar ratios ≥2.4, which causes a decrease in the number of cross-links by the formation of 2-methyl hexahydropyrimidine moieties, provides a possible explanation for this decrease in *T*_g_. Unfortunately, the small sample size of the films precludes the ability to make a reliable determination of swelling, which is associated with the number of cross-linking units. Further evidence of this phenomenon is provided by FSC, as discussed below.

Considering that the network cross-linked with DAP at an amine/βkest (feed) = 1.2 does not show any sign of the formation of aminal structures, we describe the maximum *T*_g_ for this network as 145 °C. The differences in *T*_g_ for this network with respect to the other networks cross-linked with EDO and Jeff are as high as 48 and 73 °C, respectively. As already mentioned, this result can be attributed to the more rigid structure of DAP, which contains only one propylene unit. In contrast, the ethylene oxide and propylene oxide moieties introduce greater flexibility into the cross-linked units allowing for less constrained segmental movements throughout the network. In the case of Jeff, the methyl groups of the propylene units introduce additional bulkiness causing a further reduction in *T*_g_.

### Characterizing the Vitrimeric Character of the Cross-Linked Networks with Jeff

To gain more insights into the properties of this family of materials, we have performed dynamic mechanical analysis (DMA) of the networks cross-linked with Jeff, since in this series of samples we can explore a wider temperature range, avoiding thermal degradation. A representative curve is shown in Figure S9. In all the samples we found a similar behavior; a marked drop in storage modulus in the glass transition region and a very extended plateau region at higher temperatures, with no evidence of terminal relaxation indicative of flow. Unfortunately, due to the small sample size, we were unable to determine a reliable value for the plateau modulus of these samples. Associated with this drop in modulus is a pronounced peak in the loss tangent, which can also be used to determine *T*_g_. The trend of these *T*_g_ data (shown in Figure 6b) is consistent with those obtained by DSC, although with values almost 6 °C higher. This is not surprising given the relatively high value of the frequency used in DMA (1 Hz). Moreover, the correspondence between the *T*_g_ obtained by linear measurements, as in DMA, and that obtained by measurements where the kinetic of vitrification is probed, as in DSC, is not obvious at all,^36,37^ although the former is generally greater than the latter,^38^ as in our case.

In the results presented so far, there is no clear manifestation of the vitrimeric character of these materials, which would be expected due to the dynamic nature of the covalent bonds involved in the cross-linking process. If dynamic bond exchange occurs at temperatures above *T*_g_, the DSC curves do not provide clear evidence, suggesting that the typical time scale of bond exchange would be similar to that of the segmental motions responsible for *T*_g_, i.e. the two phenomena would be strongly coupled on the time scale relevant to these measurements, that is, of the order of seconds. On the other hand, it is generally known that the segmental dynamics rate exhibits a strong non-Arrhenius temperature dependence and consequently a very high apparent activation energy, while the dynamic bond exchange rate generally follows Arrhenius behavior with a moderate activation energy.^6,14^ This situation is schematically summarized in Figure S1. From this plot, it is clear that if the coupling between the two phenomena occurs around *T*_g_, as detected by conventional DSC, a clear distinction could be detected by exploring faster rates. Based on these ideas, we studied the behavior of the samples cross-linked with Jeff using FSC, which allows identifying thermal events at experimental time scales much shorter than in standard DSC. Thanks to FSC’s ability to access heating/cooling rates as large as thousands of K s^–1^, we explored a wide range of thermal protocols, far beyond those accessible by standard DSC.^17^

A preliminary look at the calorimetric response of FSC is provided in Figure 7 for several selected systems, including the precursor polymer prior to cross-linking. Specifically, we report heating scans at 500 K s^–1^ after cooling at 1000 K s^–1^ for the cross-linked networks formed by Jeff. The heat flow rate, HF, which is proportional to the specific heat, is shown in its normalized version (eq 2)­

2 $${HF}_{norm}\left(T\right)=\frac{HF\left(T\right)-{HF}_{gl}\left(T\right)}{{HF}_{liq}\left(T\right)-{HF}_{gl}\left(T\right)}$$

where HF_gl_ and HF_liq_ are the glass and liquid heat flow rates, respectively. Although generally detected at higher temperatures with FSC than with standard DSC, we still observed a single step in the specific heat, albeit over a wider temperature interval. This was in some cases larger than 50 K, which is inconsistent with the detection of a single standard glass transition. In contrast, as shown in Figure 7, the FSC response under the same conditions for the PG-βkest precursor exhibited the typical glass transition behavior consisting of a relatively narrow heat flow rate step encompassing less than 30 K at the used high rates.

**Figure 7:**
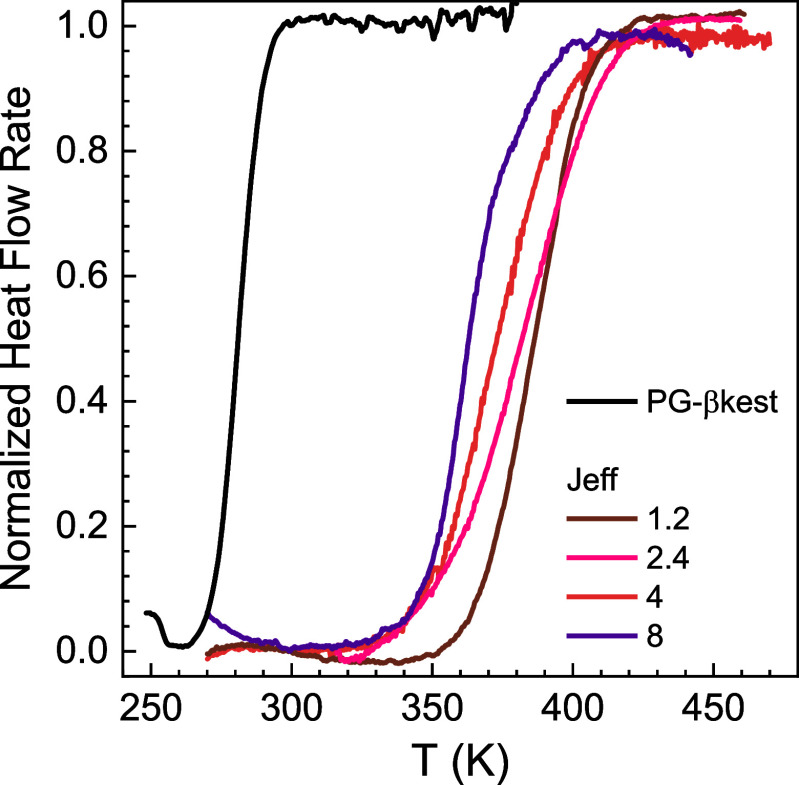
Normalized heat flow rate at 500 K s^–1^ after cooling at 1000 K s^–1^ for the networks obtained with Jeff at the indicated amine/βkest (feed) molar ratios.

A step forward in the detection of multiple thermal events is provided by thermal protocols in which the cooling rate is varied over the wide range allowed by FSC. This type of protocol is typically adopted to emphasize the presence of kinetic thermal events such as the glass transition,^39–41^ even when these are barely visible in simple heating/cooling scans at the same rate.^42^ Here, we subjected a number of studied networks to cooling rates ranging from 0.05 to 1000 K s^–1^ and assessed how the variation in cooling rates was reflected in the heating scan at 500 K s^–1^ performed immediately after. The results (Figure 8) show that heating after slow cooling results in the development of an endothermic overshoot, indicating the attainment of glassy states with low enthalpy. Importantly, on heating after cooling at the lowest rates, a bimodal overshoot is observed, or at least a very broad endotherm encompassing almost 100 K is evident.

**Figure 8:**
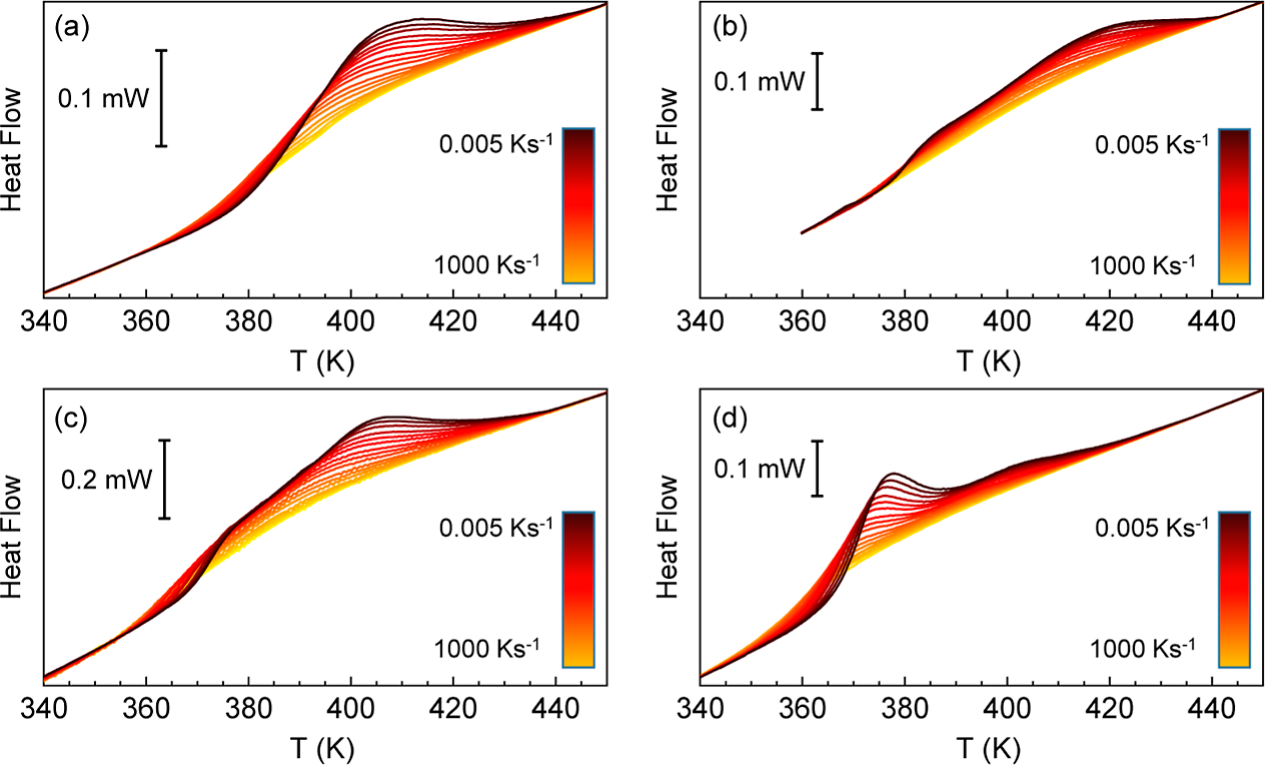
Heat flow rate temperature scans upon heating at 500 K s^–1^ after cooling at the indicated rates for samples obtained with Jeff at an amine/βkest (feed) molar ratio of (a) 1.2, (b) 2.4, (c) 4 and (d) 8.

### Unraveling the Vitrimeric Character of the Cross-Linked Networks by Fast Scanning Calorimetry

The observation of a single broad endotherm, though indicating the presence of different thermal events, does not allow to unambiguously disentangle them. Instead, this can be done in those cases where the bimodal character is more evident. To gain insight into the mechanisms underlying the presence of these two events—specifically their relation with the glass and topological transition temperatures, *T*_g_ and *T*_V_, respectively, both expected to occur in vitrimers—we have enriched our calorimetric analysis by subjecting two judiciously selected networks to increasingly complex thermal protocols. The selected samples were those obtained with Jeff at an amine/βkest (feed) molar ratio of 2.4 and 8 (hereafter named Jeff_2.4_ and Jeff_8_, respectively), which clearly exhibit two distinguishable thermal events (Figure 8). Specifically, our thermal protocol consisted of varying the heating rate after cooling at different rates. The resulting set of heating scans is presented in Figures 9 and 10, which shows that two thermal events can be detected for all investigated heating rates. Here, it is worth pointing out that the maximum heating rate for Jeff_2.4_ is 500 K s^–1^, which is lower than that of Jeff 8 (1000 K s^–1^). The reason is that the former sample has larger mass and, therefore, it exhibits significant thermal lag at 1000 K s^–1^ (Figure S10). Interestingly, the separation of both thermal events appears to be more pronounced at higher heating rates. This latter observation qualitatively indicates that the high temperature event presents a lower apparent activation energy than the low temperature event. Another interesting observation is that the comparison between both samples, obtained with different amounts of Jeff, suggests that the thermal response of the two events is unevenly distributed. Specifically, while the low temperature thermal event exhibits a large endothermic effect with respect to the high temperature one for Jeff_8_, the opposite applies for Jeff_2.4_.

**Figure 9:**
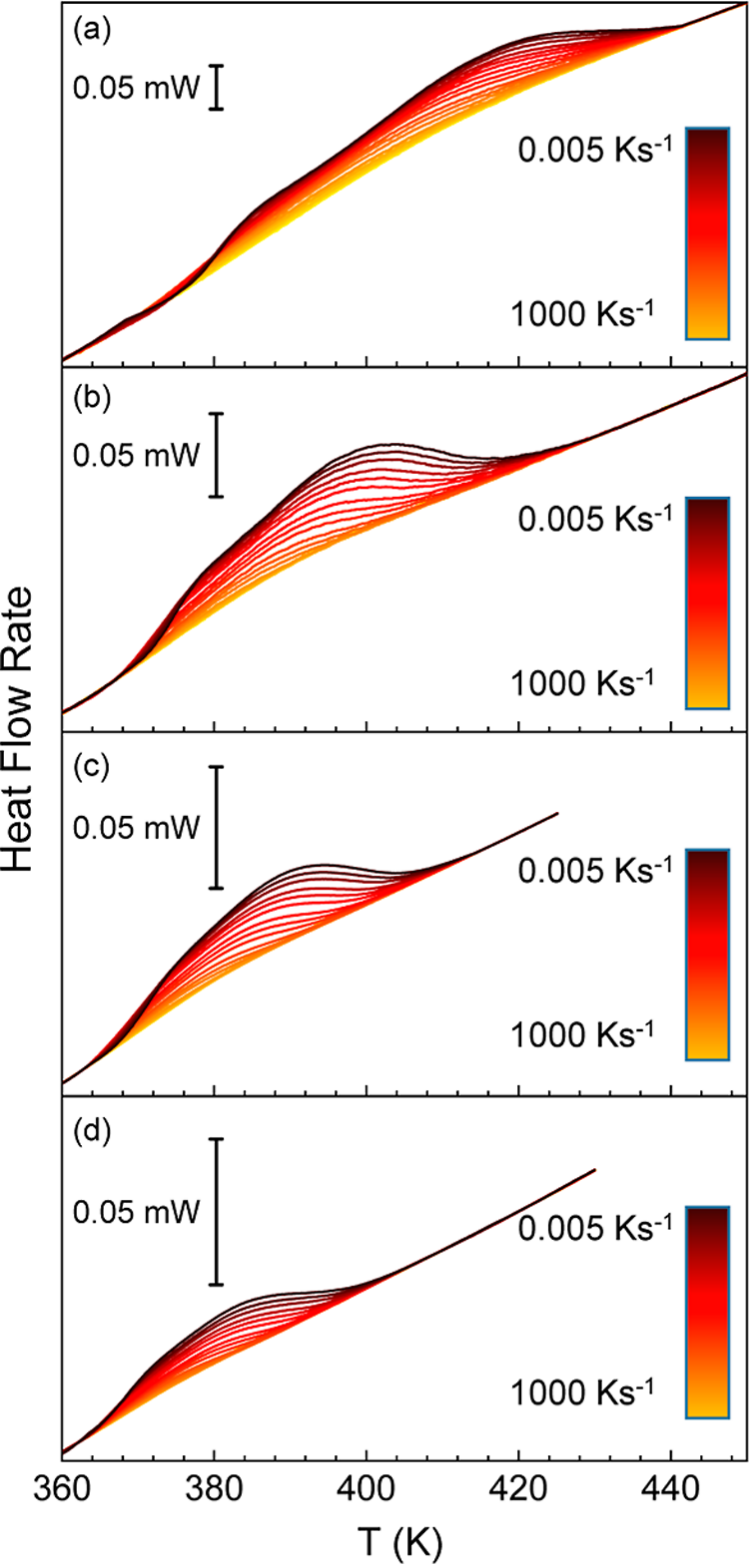
Heat flow rate scans obtained upon heating at (a) 500 K s^–1^, (b) 200 K s^–1^, (c) 100 K s^–1^ and (d) 50 K s^–1^ after cooling in a range between 0.005 and 1000 K s^–1^ for Jeff_2.4_.

**Figure 10:**
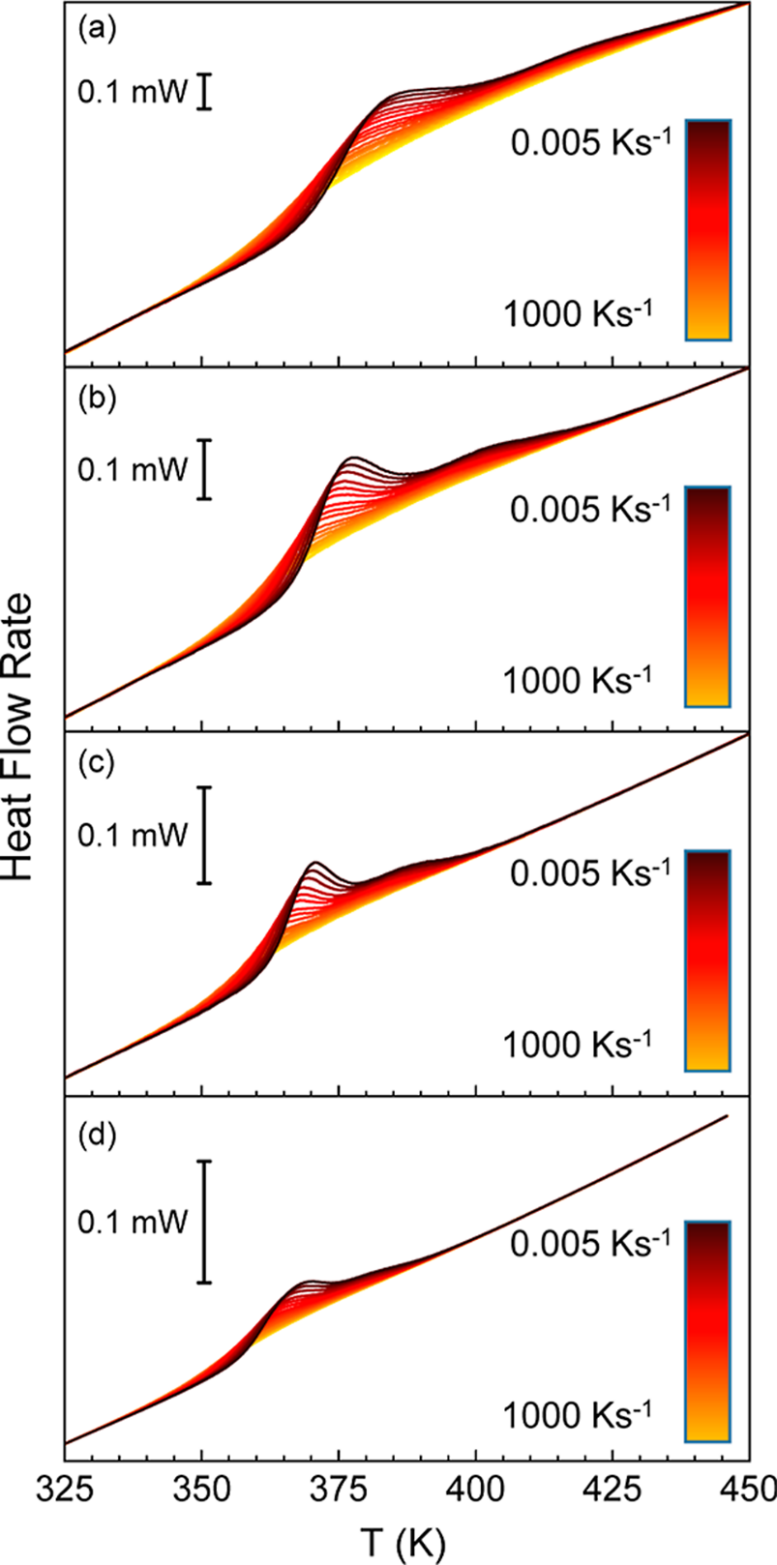
Heat flow rate scans obtained upon heating at (a) 1000 K s^–1^, (b) 500 K s^–1^, (c) 200 K s^–1^ and (d) 100 K s^–1^ after cooling in a range between 0.005 and 1000 K s^–1^ for Jeff_8_.

The endothermic excess observed when samples previously cooled at low rates are heated underlines the kinetic transformations from the glass to states where configurational degrees of freedom are progressively activated. Insights into these transformations can be obtained by identifying the temperature, *T*_p_, at which the rate of enthalpy change is maximum. While strictly speaking this corresponds to the peaks of the heat flow rate scans, inspection of Figures 9 and 10 reveals that in most cases these cannot be unambiguously separated. Consequently, to perform a simple data analysis, we assumed that the distribution of relaxation times of the two thermal events detected by FSC is temperature invariant. This implies that time–temperature superposition holds separately for each of the two events, which has been approximately verified over moderately wide temperature ranges in polymeric systems.^43,44^ Starting from these premises, information about the transformation kinetics of the observed thermal events can be obtained considering representative temperatures at which these events do not overlap. Therefore, as a proxy for *T*_p_—which for simplicity we will also refer to as *T*_p_—we have resorted to the temperatures at which the rate of change of the heat flow rate is maximum. In both cases, a criterion of nonoverlap of the two events was chosen. Specifically, for the low temperature event the rate of maximum increase in heat flow rate was chosen, while for the high temperature one the criterion was based on the rate of maximum decrease in heat flow rate before the kinetic transformation ends. In this way we minimize the superposition effect of the two thermal events on characterizing each individual contribution by selecting the standard inflection point temperature for the lower-temperature event and the endset temperature for the higher-temperature event. Thus, these temperatures are unambiguously identified where the second derivatives of the heat flow rate scans shown in Figures 9 and 10 become zero. An overview of the second derivatives of the heat flow rate scans is shown in Figure S11 for representative heating (200 K s^–1^) and cooling rates (0.005–0.1 K s^–1^).

The chosen proxy for the rates of maximum transformation at different heating rates underlined by the two *T*_p_ can be used to implement the so-called Kissinger analysis.^45,46^ The latter is widely used in thermal analysis to gain insights into the kinetic mechanisms underlying nonisothermal chemical reactions^45^ and crystallization.^47^ Within this framework, we aim to convey information about the apparent activation energies (*E*_a_) of the two thermal events underlying the nonisothermal kinetic transformations, in this case from the frozen-in glass to the high temperature viscous flow regime.^48^ The rate of kinetic transformation, *k*, can be written as (eq 3)­

3 $$k=k_{0}\exp\frac{{-E}_{a}}{RT}$$

where *k*_0_ is a pre-exponential factor, *R* is the gas constant, and *E*_a_ *is* apparent activation energy of the molecular mechanism supporting the kinetic transformation under study.

In the kinetic transformation from an initial state to a final state, e.g. from glass, with degree of transformation *X* = 1, to liquid, with *X* = 0, in the most general case the transformation rate is related to *k* as (eq 4)­

4 $$\frac{dX}{dt}={k\left(1-X\right)}^{n}$$

where *n* is the order of the kinetic transformation.

Considering the criterion of maximum enthalpy transformation, at *T*_p_ the first derivative of the rate of change of the kinetic transformation must be zero (eq 5)­

$$\frac{\frac{dX}{dt}}{dt}=k_{0}\exp\left(\frac{{-E}_{a}}{RT_{p}}\right)\frac{{-E}_{a}}{RT_{p}^{2}}{\left(1-X\right)}^{n}\frac{dT_{p}}{dt}-n{\left(1-X\right)}^{n-1}\exp\left(\frac{{-E}_{a}}{RT_{p}}\right)\frac{dX}{dt}=0$$ 5

In most cases the exponent *n* is close to unity^45^ and, therefore, *n*(1 – *X*)^*n*−1^ = 1. Rearranging eq 5 and writing the heating rate d*T/*d*t* = β, we obtain (eq 6)­

6 $$\ln\frac{\beta }{T_{p}^{2}}=-\frac{E_{a}}{RT_{p}}+c$$

where *c* is a temperature independent parameter, although its invariance is strictly valid only if *E*_a_ of the thermal event under consideration is also temperature independent. In the case of our study, this is not strictly true in the case of the glass transition,^48,49^ generally associated with the polymer segmental motion, whose *E*_a_ is temperature dependent. However, given the relatively small temperature range explored in our study, eq 6 can still be considered valid.

According to eq 6, the slope of ln­(β*/T*_p_^2^) as a function of 1*/T*_p_ allows obtaining *E*_a_ of the molecular mechanism mediating the kinetic transformation process. The correlation established by eq 6 is presented in Figure 11 for both Jeff_2.4_ and Jeff_8_, showing the *T*_p_ data of the two observed thermal events after cooling and heating at different heating rates. It should be noted that the fit via eq 6 for the high temperature event data was restricted to the three highest heating rates, since the lowest rate exhibits a clear deviation toward a higher *E*_a_, a fact that will be discussed later. As can be observed for both systems, the high temperature thermal event has a lower *E*_a_ compared to the low temperature event. Furthermore, a comparison of the Kissinger plots of the two systems shows that Jeff_8_ generally has lower transformation temperatures than Jeff_2.4_. Although in the same range, the *E*_a_ of the two events for Jeff_2.4_ appear to be slightly larger than those for Jeff_8_.

**Figure 11:**
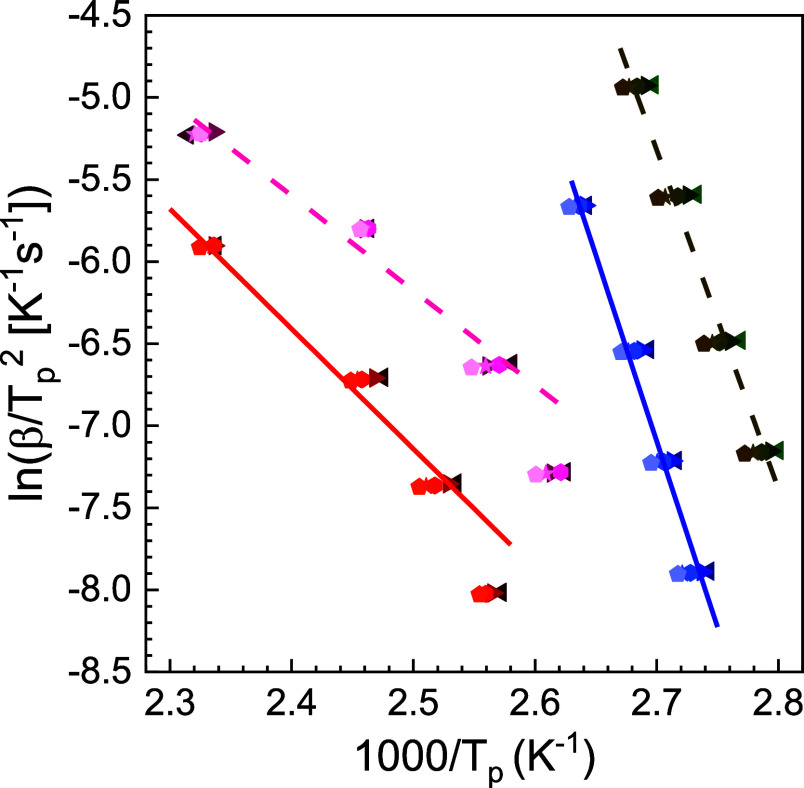
Kissinger plot of the thermal transformation events observed in Jeff_2.4_ (solid, red and blue) and Jeff_8_ (dash, pink and khaki) after cooling at the following rates: 0.1 (left triangles), 0.05 (right triangles), 0.02 (hexagons), 0.01 (stars) and 0.005 (pentagons) K s^–1^. The straight lines are the fits to eq 6, which yields the following apparent activation energies for the higher and lower temperature events, respectively: *E*_a_ = 60 ± 5 kJ mol^–1^ and *E*_a_ = 190 ± 5 kJ mol^–1^ for Jeff_2.4_ and *E*_a_ = 50 ± 5 kJ mol^–1^ and *E*_a_ = 170 ± 5 kJ mol^–1^ for Jeff_8_.

The values of *E*_a_ of the two transformation kinetics observed in both Jeff_2.4_ and Jeff_8_ can be understood complementing this information with that obtained from experiments conducted in the linear regime.^37^ This is done employing step response analysis.^30,31^ Figure 12a,b show the temperature and frequency dependent normalized reversing specific heat, *C*_p,rev_^*N*^, which is approximately equal to the normalized real part of the complex specific heat.^41^ A single relatively narrow step in *C*_p,rev_^*N*^ is observed. This can be unambiguously attributed to spontaneous fluctuations of the polymer segments associated with the glass transition, as commonly observed in all types of glass-forming systems.^50,51^ The temperature dependence of the typical time scale of these fluctuations: τ = 1/(2πf), taken from the temperature of maximum inflection of *C*_p,rev_^*N*^, is shown in Figure 12c. As expected, this relaxation time exhibits a marked temperature dependence with super-Arrhenius temperature behavior, which can be described by the Vogel–Fulcher–Tammann (VFT) empirical equation^52–54^ (gray line): τ­(*T*) = τ_0_exp­(*B*/(*T* – *T*_0_)). As noted above, the apparent activation energy obtained from the VFT equation is temperature dependent and is given by $E_{VFT}=T^{2}B/{\left(T-T_{0}\right)}^{2}$ . To verify how the two kinetic processes identified by FSC are related to the polymer segmental dynamics, we have analyzed the temperature dependence of *T*/*E*_a_^1/2^ vs *T*, which for the VFT case would result in a linear plot: *T*/*E*_VFT_^1/2^ = (*T* – *T*_0_)/*B* ^1/2^. Figure 13 shows such a comparison, from which it is clear that the low-temperature thermal event agrees well with the expectation from the VFT equation, while, in contrast, the data for the high-temperature kinetic process are far above the VFT line, indicating a significantly lower apparent activation energy as expected for a vitrimeric-related phenomenon. It should be noted that the underlying molecular mechanism for the high-temperature kinetic transformation remains invisible in the calorimetric characterization in the linear regime. This can be attributed to the two following reasons: (i) weakly activated thermal events are hardly visible due to the fact that they cover a wide temperature range;^44^ (ii) the degrees of freedom activated by the high temperature event are limited, making the associated specific heat step small.

**Figure 12:**
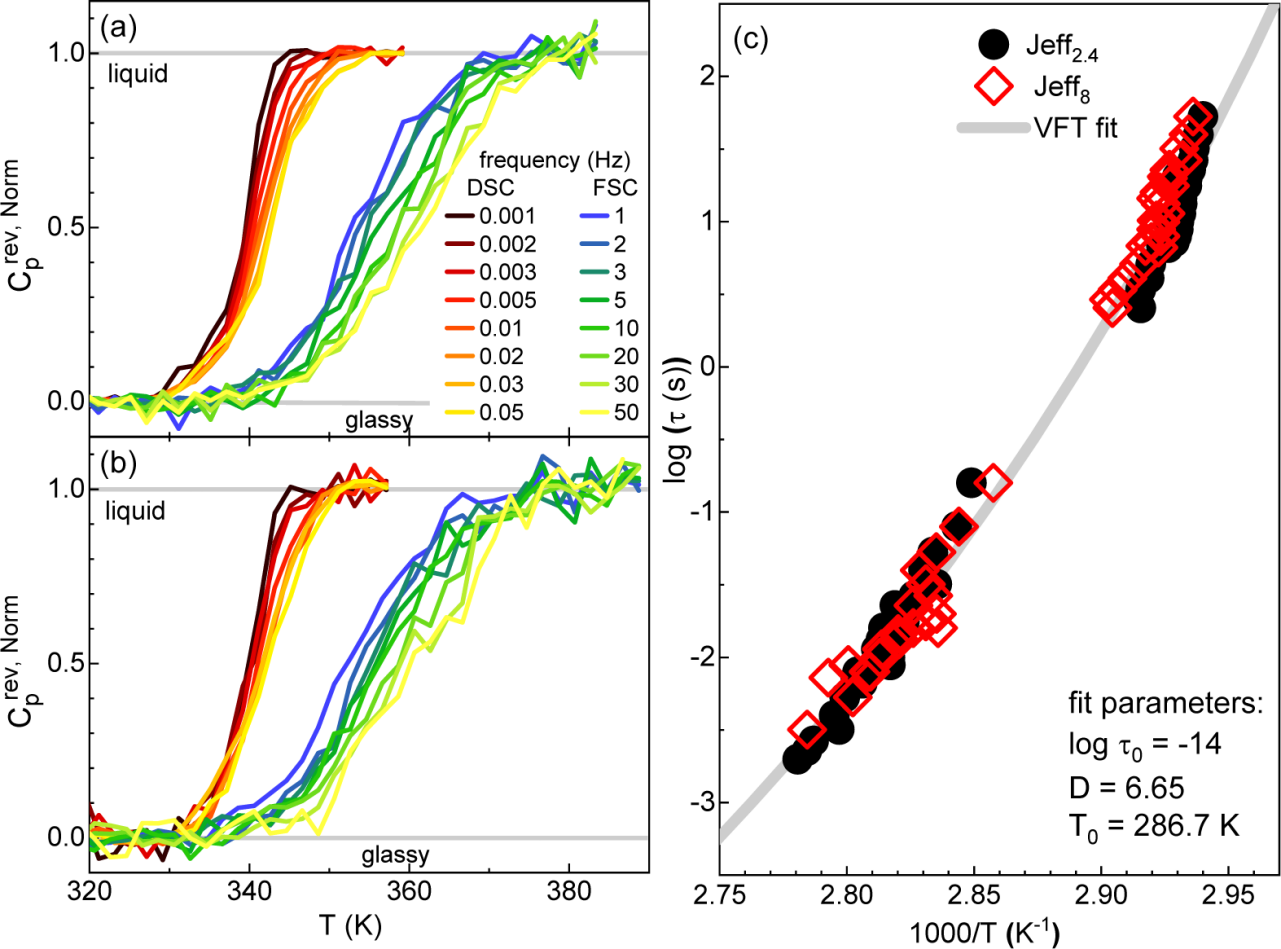
Normalized reversing specific heat as a function of temperature at different frequencies obtained from standard DSC and FSC for (a) Jeff_2.4_ and (b) Jeff_8_. (c) Temperature dependence of the relaxation time obtained from panels (a,b) at the temperature of maximum inflection of the normalized reversing specific heat. The line in panel (c) is the VFT fit with parameters given in the legend.

**Figure 13:**
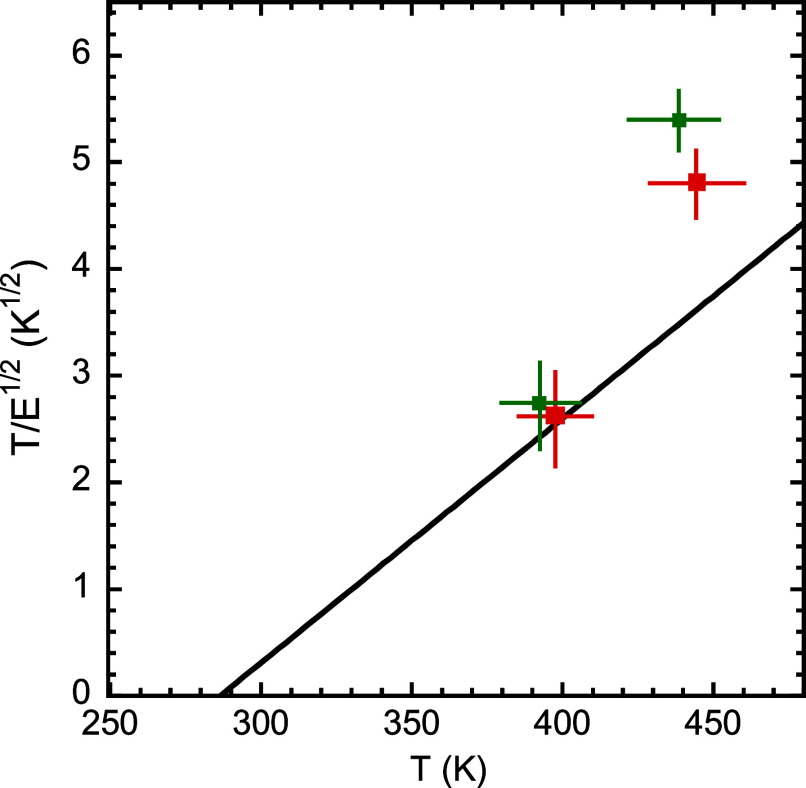
Comparison of the apparent activation energies obtained from FSC for the two kinetic processes for Jeff_2.4_ (green) and Jeff_8_ (red) with that corresponding to the VFT equation describing the relaxation times determined in the linear regime. The size of the crosses corresponds to the estimated uncertainties.

As a final complementary test of the vitrimeric character of the present networks, we performed experiments using parallel plate viscometry,^55^ which records changes in sample thickness as a function of time while a constant small vertical force is applied. In this way, we found clear evidence of slow flow at temperatures above 370 K. Figure 14 shows that the high viscosity values obtained, around 10^11^ Pa s, follow an Arrhenius temperature dependence with an activation energy of about 100 kJ/mol, a value intermediate between those found for the two kinetic phenomena detected by FSC.

**Figure 14:**
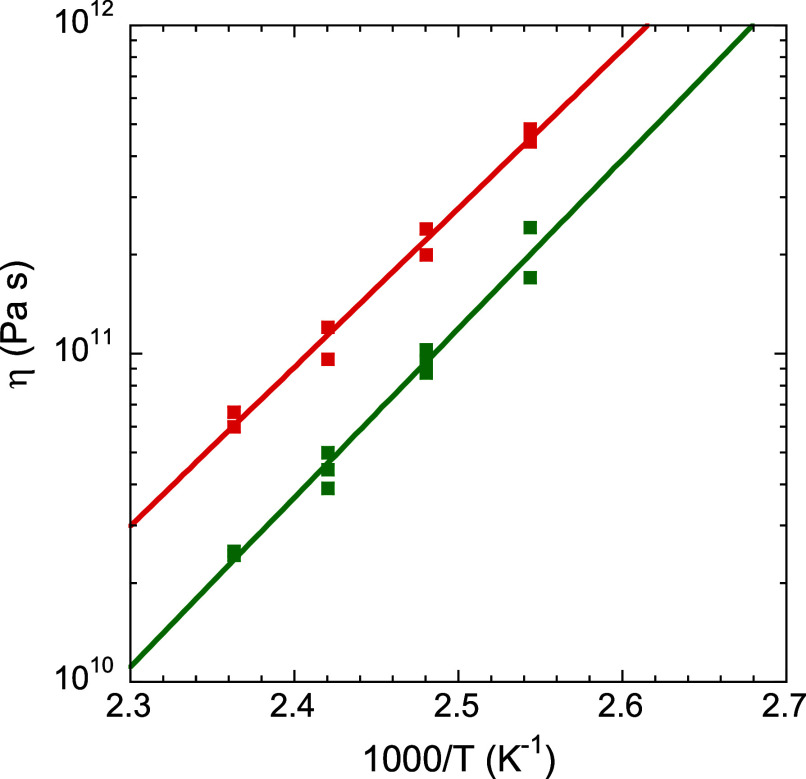
Arrhenius plot of the viscosity as determined by parallel plate viscometry for Jeff_2.4_ (green) and Jeff_8_ (red). Several experimental data points at each temperature are shown to illustrate typical uncertainties. The solid lines correspond to the corresponding Arrhenius fits.

Overall, our results on the kinetic transformation from frozen-in glass to fully relaxed network indicate that it occurs via a low and a high temperature event, the former being completely coupled to the polymer segmental relaxation responsible for the glass transition. The high temperature event has a lower apparent activation energy, which is consistent with a reversible covalent bond exchange reaction.^14^ The latter process is generally described as occurring in two steps: (i) approach of two active sites by segmental diffusion and; (ii) bond exchange once these active sites are brought into close contact.^56^

In the high temperature regime, in the case of Jeff_2.4_ and Jeff_8_ above ∼390 and 400 K, respectively (see Figure 12), the segmental diffusion would exhibit a time scale much shorter than that responsible for bond exchange. As a result, the low activation energy in this regime is representative of the bond exchange process alone, that is, the slow step of the overall bond exchange reaction. At lower temperatures, segmental diffusion rapidly becomes slower, thereby making the overall covalent bond exchange reaction to be controlled by both steps. This picture is fully compatible with our results on how the kinetic transformation of the high temperature event takes place, which shows an increasing apparent activation energy at low temperatures (see the data points deviating from the Kissinger fit in Figure 11). At lower temperatures, the overall process of the reversible covalent bond exchange reaction is expected to be completely controlled by the ultraslow segmental relaxation under these conditions. As a result, this process will be indistinguishable from segmental relaxation.^15,56^ In other words, the two stages of the bond exchange process, that is, diffusion and chemical bond exchange, become coupled in the low-temperature regime, leading to an apparent increase in activation energy. The latter observations may explain why only a relatively narrow specific heat step is observed by standard DSC, considering that the latter technique, due to the long accessible observation times, i.e. much lower heating/cooling rates compared to FSC, actually explores the low temperature regime. This is found only for very large separation between segmental relaxation and bond exchange reaction time, which is rarely observed unless nanophase separation takes place.^57,58^ Standard DSC has previously been shown to provide convincing evidence of the two thermal events only in some rare cases.^15^

The activation energy of temperature dependent viscosity can be compared with that found by FSC. As can be seen, the activation energy of viscosity obtained from Figure 14 has a value somewhat larger that of the high temperature bond exchange kinetics: 100 kJ mol^–1^ vs 50 and 60 kJ mol^–1^ for Jeff_2.4_ and Jeff_8_, respectively. These results indicate that the viscosity of the investigated vitrimers is only partly controlled by the bond exchange kinetics. A review of previous results in the literature indicates that this is the case for some vitrimeric systems, while for others bond exchange fully control the viscosity.^15^

An important point worth of discussion regards the difference in transformation kinetics and viscosity between Jeff_2.4_ and Jeff_8_. Although these samples exhibit an amine/beta-ketoester ratio of approximately 1, both were prepared with very different excess amounts of amine (2.4 for Jeff_2.4_ and 8 for Jeff_8_). At these amine concentrations, the formation of “dangling chains” is highly favored. Once the curing is performed at 85 °C, followed by the removal of excess amine, the enamines are reformed through reaction with the free amines from the “dangling chains”, which results in the formation of diamines connected on both sides. This results in a network that is totally cross-linked both intra- and intermolecularly, where each beta-ketoester group corresponds to one amine (amine/β-ketoester molar ratio ∼1). However, it is probable that the Jeff_8_ sample contains a higher amount of amines that have initially penetrated into the interior of the branched structure compared to the Jeff_2.4_ sample. This will conduct to a distribution of cross-linked units that differs between Jeff_2.4_ and Jeff_8_, as suggested by the narrower *T*_g_ range and the more pronounced separation from the *T*_v_ in Jeff_8_ compared to Jeff_2.4_. Therefore, we conclude that the amount of amine used in the preparation of the cross-linked networks is a pivotal parameter for obtaining more homogeneous materials.

These considerations are also relevant to the relative intensity of the glass transition and vitrimeric kinetics obtained from FSC. Specifically, a comparison of panels (b) and (d) in Figure 8 indicates that the event related with the glass transition is more intense than the vitrimeric transformation in Jeff_8_, whereas the opposite applies for Jeff_2.4_. Tentatively, this can be rationalized by considering that the dynamic bond exchange in Jeff_8_ predominantly occurs intradendrimerically whereas that in Jeff_2.4_ occurs more interdendrimerically. This makes the macroscopic FSC enthalpic variation in Jeff_8_ milder than in Jeff_2.4_. This interpretation is consistent with the increased viscosity observed for Jeff_8_ compared to Jeff_2.4_ (see Figure 14).

In the DAP series the amount of amine was also a relevant parameter. An excess of DAP led to the formation of non-cross-linked aminal structures as shown by decreasing *T*_g_ values (Figure 6b) and the appearance of bands at 1739 and 1238 cm^–1^ in the FTIR data (Figure 5). Examining the FSC data of DAP_2.4_ and DAP_8_ (Figure S12) using similar heating and cooling protocols as the Jeff series revealed clear differences between the two sample preparations. DAP_2.4_ exhibited two thermal events related to glass transition and bond exchange, while DAP_8_ showed only one event related to glass transition. This confirms the loss of bond exchange events in DAP_8_ due to the formation of aminal structures.

## Summary

Cross-linked vitrimeric networks formed by dynamic enamine bonds were obtained through the reaction of β-ketoester-functionalized branched polyglycerol and diamines. This reaction achieved the maximum possible number of cross-links. The physical properties of these materials were found to be highly dependent on the type and amount of amine added relative to the amount of β-ketoesters, even after excess amine was removed. First, changing the type of amine alters the *T*_g_, resulting in lower *T*_g_ for networks containing oligomeric and flexible diamines (e.g., Jeff and EDO) than for networks with smaller diamine molecules (e.g., DAP). We hypothesize that a greater number of intradendrimeric bonds are formed when there is a greater excess of amine during preparation. However, with a smaller excess, interdendrimeric bonds appear to be favored. Finally, DAP is not recommended for forming covalent adaptable networks because it forms aminal groups that impede cross-linking between chains.

For the first time, the FSC was used to evaluate the *T*_v_ of cross-linked networks. Furthermore, we were able to determine the activation energy of the bond exchange using a protocol of different heating and cooling rates in the FSC that separates this process from the *T*_g_. As expected, the activation energy obtained from the bond exchange was much lower, in the range of 50–60 kJ/mol, than the apparent activation energy of the glass transition, in the range of 170–190 kJ/mol.

Our FSC protocol, here developed, can be extended to the detection of *T*_v_ and the study of the bond exchange kinetics of a number of vitrimeric networks, highlighting the versatility of this approach in characterizing dynamic covalent materials. By enabling rapid and precise thermal analysis, the method provides critical insight into the temperature-dependent kinetic processes and the influence of network composition, and cross-link density on the vitrimeric behavior.

## Supplementary Material



## Notes

Tables of composition (composition in the feed and elemental analysis results), supporting equations, NMR and FTIR spectra, TGA and DMA data, second derivative of FSC heat flow and DFT calculation results (PDF)
