# Supplementary material for: Vitrimeric Behavior Revealed by Fast Scanning Calorimetry in Branched Polyglycerol Networks Cross-Linked by Reversible Enamine Bonds
Source: Macromolecules. 2025 Sep 3;58(18):9993–10006. doi: 10.1021/acs.macromol.5c01560 (PMC12461918; doi:10.1021/acs.macromol.5c01560)
Supplement: Supplementary file 1 [file ma5c01560_si_001.pdf]

Vitrimeric behavior revealed by fast scanning calorimetry in branched polyglycerol networks crosslinked by reversible enamine bonds

Vasiliki Maria Stavropoulou,‡[a,b] Marta Aldecoa-Ortueta,‡[a,b] Ester Verde-Sesto,[a,c] Valerio Di Lisio,[a,d] Anabel Lam,[d,e] José A. Pomposo,[a,b,c] Angel Alegría,[a,b] Daniele Cangialosi,*[a,d] Fabienne Barroso-Bujans*[a,b,c,d]

[a]Materials Physics Center (CFM-MPC), CSIC-UPV/EHU, Paseo Manuel Lardizábal 5, 20018 Donostia−San Sebastián, Spain

[b]PMAS, Faculty of Chemistry, University of the Basque Country (UPV/EHU), Paseo Manuel Lardizábal 3, 20018 Donostia−San Sebastián, Spain

[c]IKERBASQUE - Basque Foundation for Science, Plaza Euskadi 5, 48009, Bilbao, Spain

[d]Donostia International Physics Center (DIPC), Paseo Manuel Lardizábal 4, 20018 Donostia−San Sebastián, Spain

[e]Zeolites Engineering Laboratory, Institute of Materials Science and Technology (IMRE), University of Havana, 10400, La Habana, Cuba

Table of Contents

1. Vitrimeric and segmental dynamics 2
2. Synthesis of cross-linked networks with diamines 2
3. Composition 3
4. Structural characterization 4
5. DFT calculations 9
References 10

1. Vitrimeric and segmental dynamics

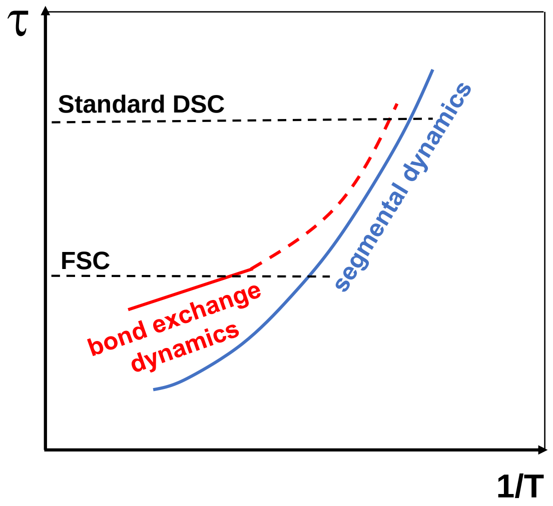

Figure S1. Scheme showing the mild Arrhenius temperature dependence of dynamic bond exchange typical time scale as opposed to the strong super-Arrhenius dependence of segmental relaxation time scale.

2. Synthesis of cross-linked networks with diamines

Table S1. Networks crosslinked with DAP.

		Vial 1		Vial 2	
**Sample**	**DAP / βkest (mol / mol)**	**$m_{PG\text{-}\beta kest}$ (mg)**	**V_{THF} (mL)**	**V_{DAP} (μL)**	**V_{THF} (μL)**
$DAP_{0.6}$	0.6	86	9.6	12	144
$DAP_{1.2}$	1.2	83	9.6	24	288
$DAP_{2.4}$	2.4	81	9.6	48	576
DAP_{3}	3.0	53	5.4	34	405
DAP_{4}	4	57	5.4	45	540
DAP_{8}	8	51	5.4	90	1080

Table S2. Networks crosslinked with Jeff.

		Vial 1		Vial 2	
**Sample**	**Jeff / βkest (mol / mol)**	**$m_{PG\text{-}\beta kest}$ (mg)**	**V_{THF} (mL)**	**V_{Jeff} (μL)**	**V_{THF} (μL)**
$Jeff_{0.6}$	0.6	78	9.6	35	144
$Jeff_{1.2}$	1.2	87	9.6	70	288
$Jeff_{2.4}$	2.4	84	9.6	140	576
$Jeff_{3}$	3.0	52	5.4	109	405
$Jeff_{4}$	4	53	5.4	147	601
$Jeff_{8}$	8	53	5.4	291	1200

Table S3. Networks crosslinked with EDO.

		Vial 1		Vial 2	
**Sample**	**EDO / βkest (mol / mol)**	**$m_{PG\text{-}\beta kest}$ (mg)**	**V_{THF} (mL)**	**V_{EDO} (μL)**	**V_{THF} (μL)**
$EDO_{0.6}$	0.6	79	9.6	21	144
$EDO_{1.2}$	1.2	85	9.6	42	288
$EDO_{2.4}$	2.4	86	9.6	84	576
EDO_{4}	4	47	6.0	88	600
EDO_{8}	8	46	6.0	175	1200

3. Composition

If all the amine functionalities of 1,3-diaminopropane (DAP) form an enamine group in the crosslinked networks, then the decimal amount of nitrogen, N, in the sample is defined by Eq. S1. The 2-methyl hexahydropyrimidine moieties formed in some samples were not included in the formula to simplify the analysis.

$$N = \frac{xM_N}{x(8.5M_C+13M_H+3M_O+1M_N)+y(7M_C+10M_H+4M_C)+z(3M_C+6M_H+2M_O)} \quad \text{Eq. S1}$$

where, M_C, M_H, M_O and M_N are the atomic masses of carbon, hydrogen oxygen and nitrogen, respectively. According to the degree of functionalization of PG with βkest functions found by ^{1}H NMR: z = 0.17 and $x + y = 0.83$. "z" is the fraction of non-functionalized hydroxyl groups of polyglycidol, "x" is the fraction of enamine groups and "y" is the fraction of non-reacted βkest groups.

Then, reformulating Eq. S1, "x" can be calculated by using Eq. S2, where N is the decimal amount of nitrogen determined experimentally and reported in Table S4 [Nitrogen (wt%)/100].

$$x = \frac{144N}{14-19N} \quad \text{Eq. S2}$$

Similarly, the amount of nitrogen, N in the crosslinked networks formed by PG-βkest and 2,2′-(ethylenedioxy)bis(ethylamine) (EDO) is defined by Eq. S3 and "x" by Eq. S4 and that formed by PG-βkest and Jeffamine D230 (Jeff) is defined by Eq. S5 and "x" by Eq. S6.

$$N = \frac{xM_N}{x(10M_C+16M_H+4M_O+1M_N)+y(7M_C+10M_H+4M_C)+z(3M_C+6M_H+2M_O)} \quad \text{Eq. S3}$$

$$x = \frac{144N}{14-56N} \quad \text{Eq. S4}$$

$$N = \frac{xM_N}{x(12.25M_C+20.5M_H+4.25M_O+1M_N)+y(7M_C+10M_H+4M_C)+z(3M_C+6M_H+2M_O)} \quad \text{Eq. S5}$$

$$x = \frac{144N}{14-91.5N} \quad \text{Eq. S6}$$

Finally, to determine the mole equivalents of amine per mol of βkest in the sample, Eq. S7 was employed.

$$\frac{\text{amine}}{\beta\text{kest(mol)}} = \frac{x}{x+y} \quad \text{Eq. S7}$$

Table S4. Amount of nitrogen in the polymer networks determined by elemental analysis.

amine / βkest (mol/mol) Feed	Nitrogen (wt%) Sample DAP	Nitrogen (wt%) Sample EDO	Nitrogen (wt%) Sample Jeff
0.6	3.81	5.43	3.80
1.2	5.87	6.46	5.06
2.4	6.82	6.45	5.08
3.0	6.57	-	5.10
4.0	6.78	6.53	5.44
8.0	6.6	6.51	5.15

4. Structural characterization

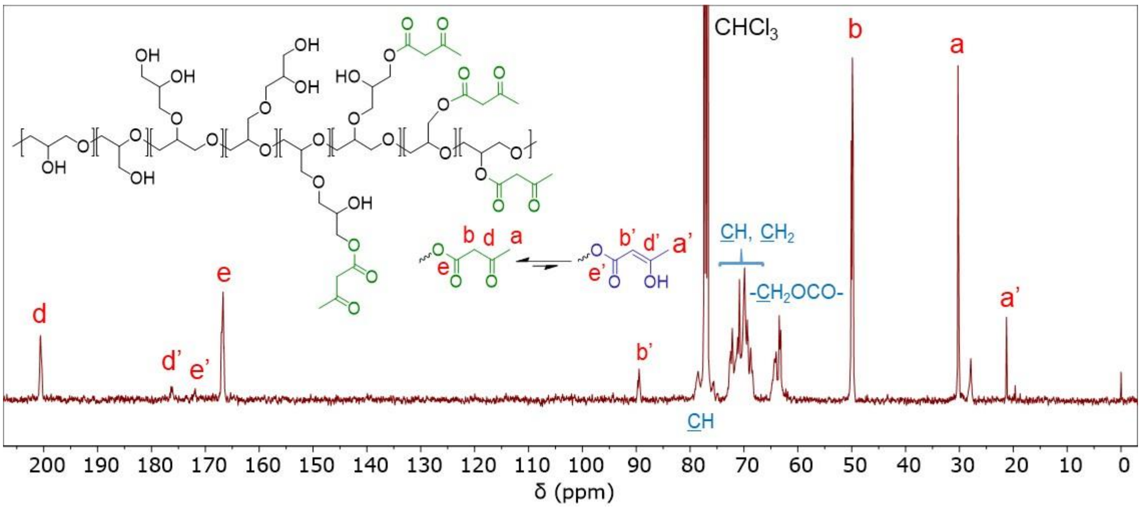

Figure S2. ^{13}C NMR spectrum (in $CDCl_3$) of PG-βkest.

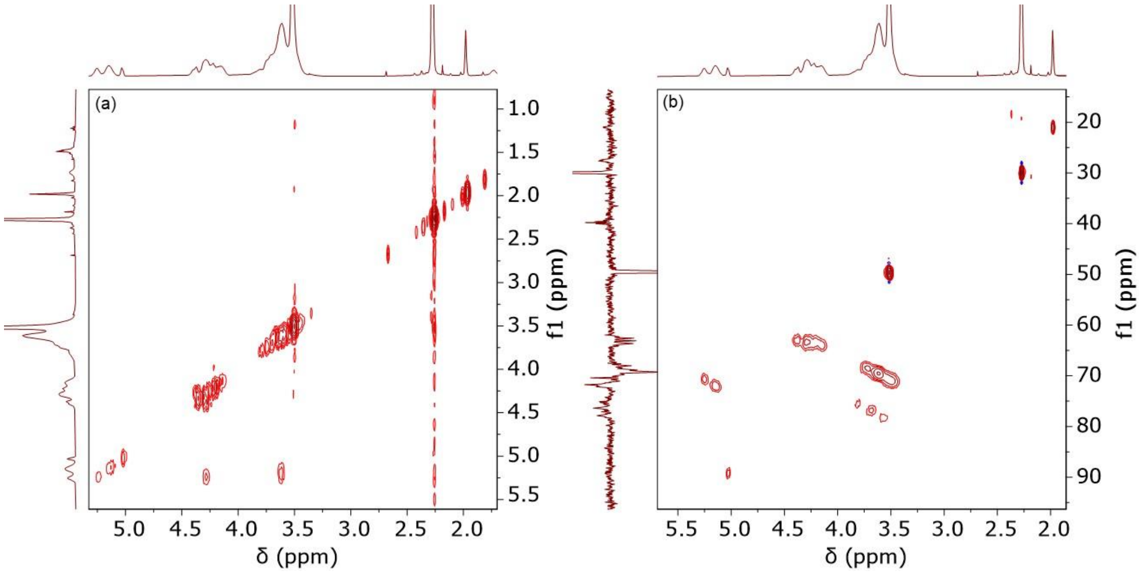

Figure S3. (a) COSY and (b) HSQC-DEPT 135° spectra (in $CDCl_3$) of PG-βkest.

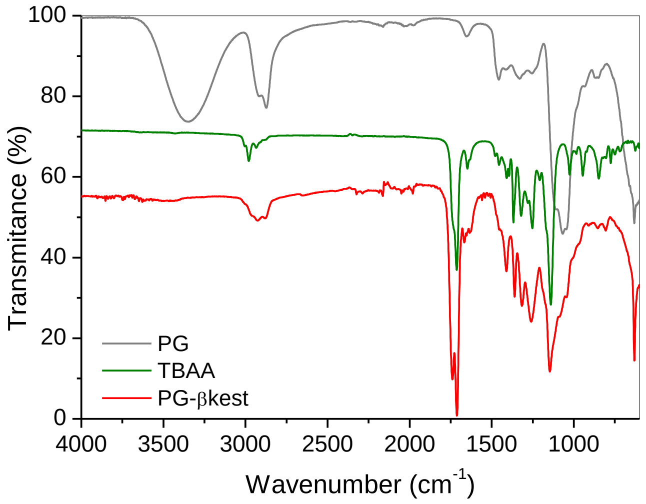

Figure S4. FTIR-ATR spectra vertically shifted for illustrative purposes.

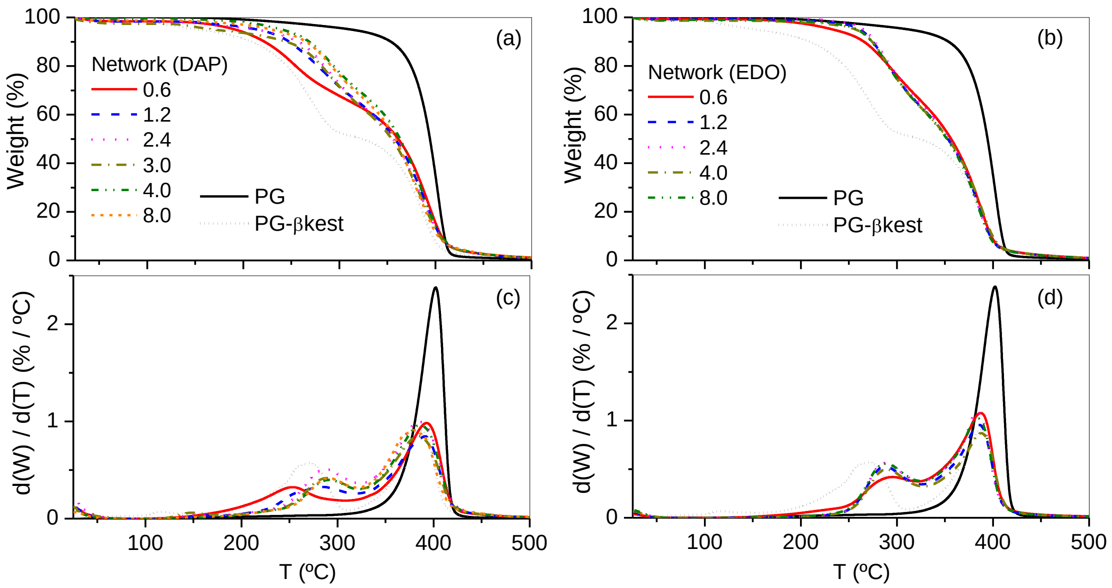

Figure S5. TGA data recorded at 10 ºC/min under nitrogen atmosphere of PG, PG-βkest and crosslinked networks obtained by reaction of (a, c) PG-βkest with DAP and (b, d) PG-βkest with EDO using different amine / βkest molar ratios in the feed (from 0.6 to 8.0). Top: Weight loss. Bottom: First derivative of weight with respect to temperature.

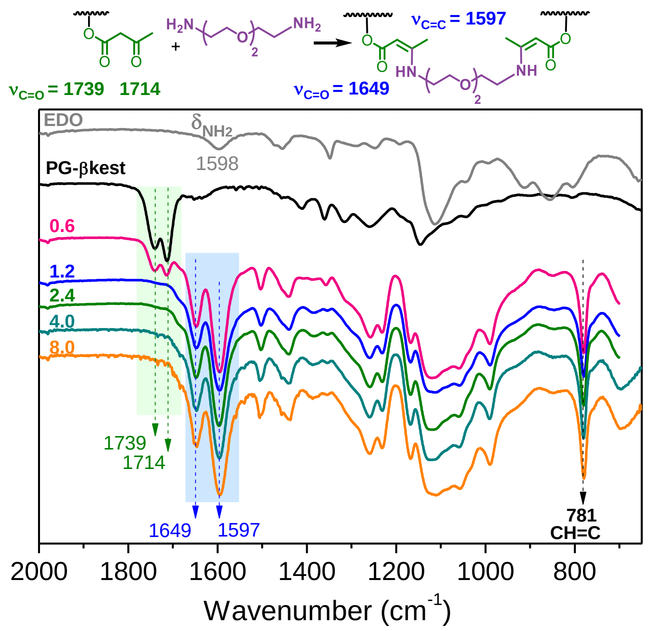

Figure S6. FTIR spectra of the crosslinked networks formed by PG-βkest and EDO obtained with different amine / βkest molar ratios in the feed (from 0.6 to 8.0).

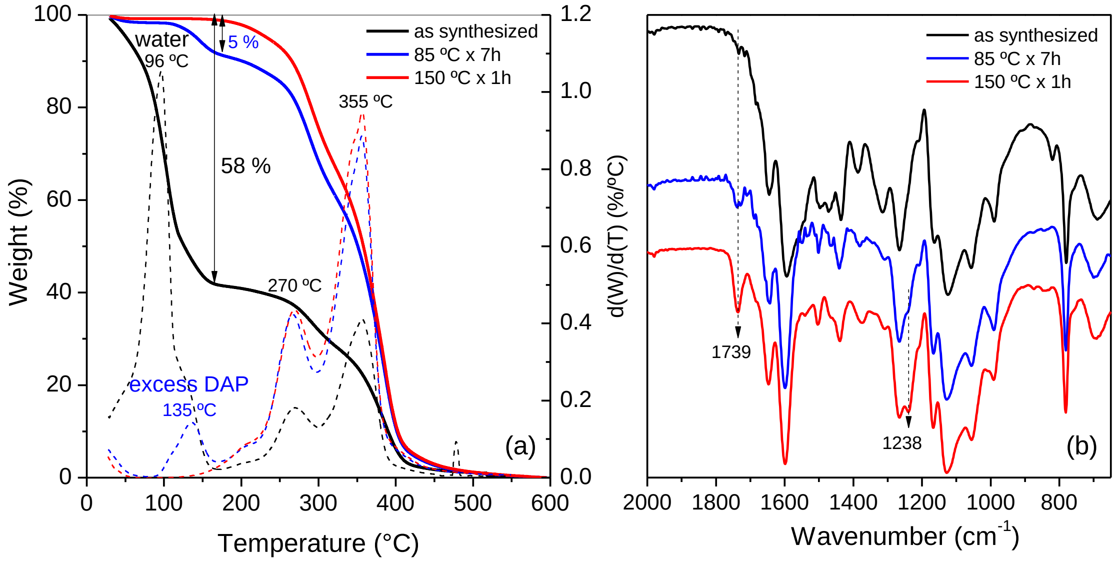

Figure S7. (a) TGA and (b) FTIR spectra of the crosslinked networks formed by PG-βkest and DAP obtained with amine / βkest = 4. Sample "as synthesized" was not heated nor purified. A piece of this sample was heated at 85 ºC for 7 h in vacuum showing unsuccessful removal of DAP while successful removal of water. Another piece of sample was heated at 150 ºC for 1 h in vacuum proving the successful removal of DAP and water. Absorption bands appeared at 1739 and 1238 cm^{-1} upon both thermal treatments, indicating the formation of 2-methyl hexahydropyrimidine moieties.

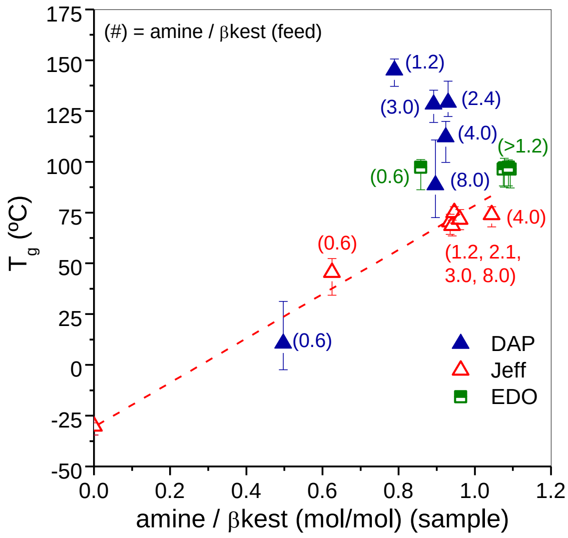

Figure S8. T_g of crosslinked networks as a function of amine / βkest molar ratios in the sample, determined by DSC.

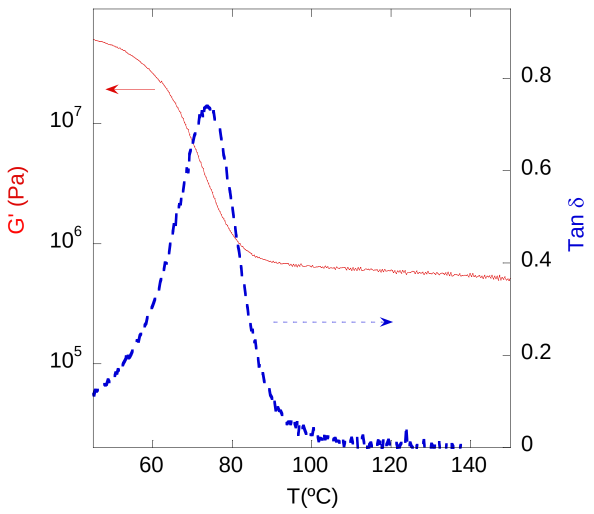

Figure S9. Representative DMA data for $Jeff_{2.4}$ obtained at 0.1% strain at 1 rad/s frequency.

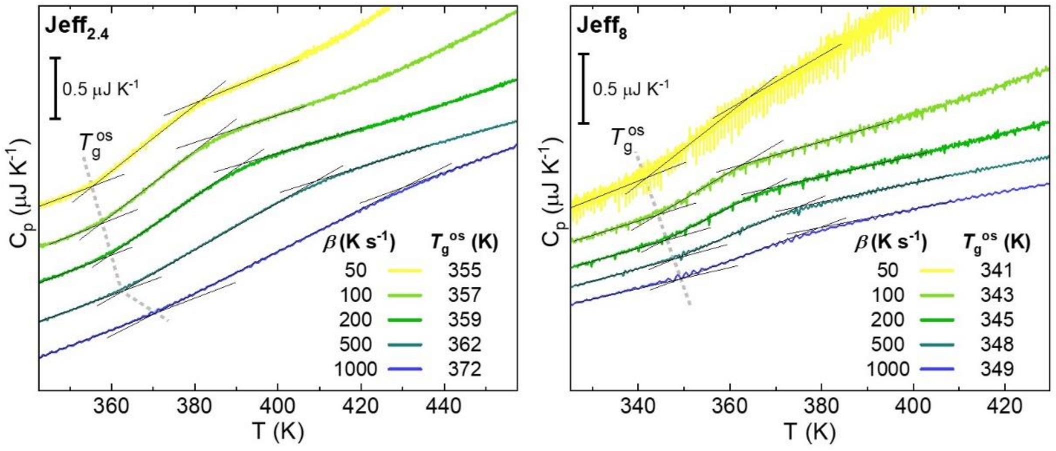

Figure S10. Heating scans for $Jeff_{2.4}$ (left) and $Jeff_8$ (right) at different rates showing the significant thermal lag in the former at 1000 Ks^{-1}.

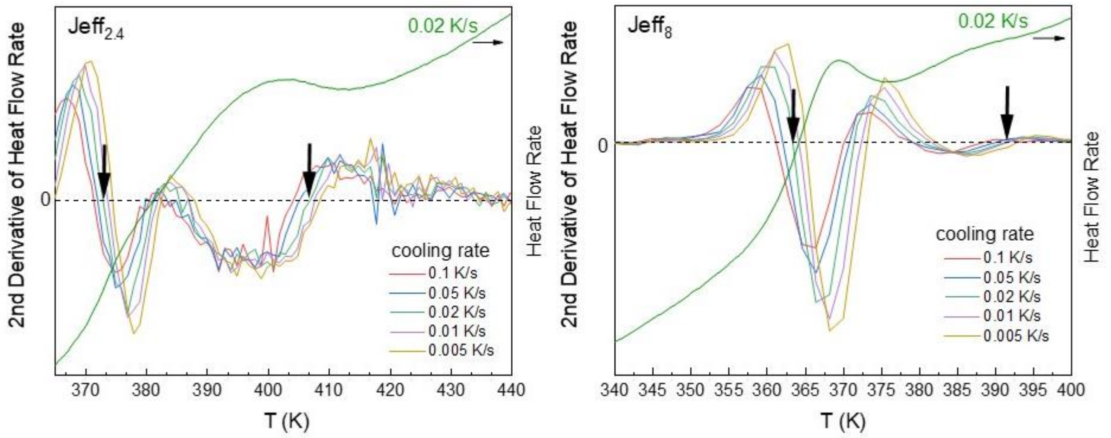

Figure S11. Examples of second derivatives of specific heat scans for $Jeff_{2.4}$ (left) and $Jeff_8$ (right) at 200 K/s.

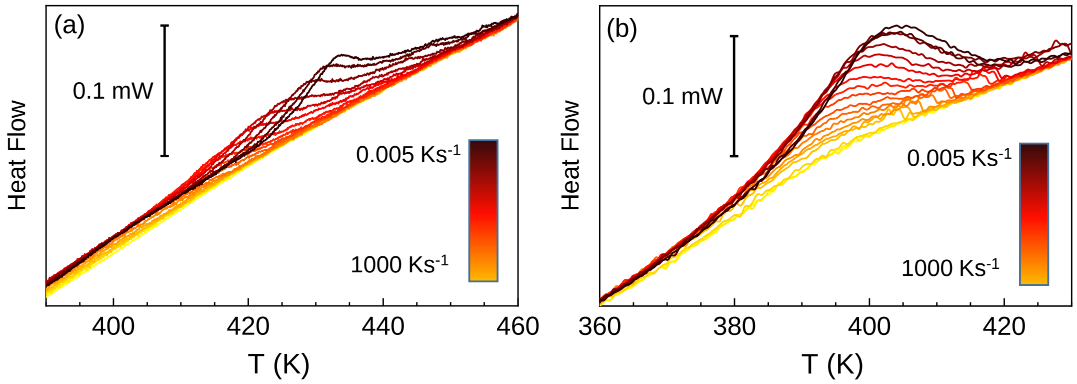

Figure S12. Heat flow rate temperature scans upon heating at 500 Ks^{-1} after cooling at the indicated rates for samples obtained with DAP at an amine / βkest (feed) molar ratio of (a) 2.4 and (b) 8.

5. DFT calculations

The enthalpies of aminal and enamine model compounds formed by DAP, EDO and Jeff were calculated by performing DFT-D3 geometry optimizations using the Gaussian 16 program.[1] The DEF2TZVP basis set[2-3] and the TPSS exchange-correlation functional[4] were used. To simulate the environmental effect, a dielectric constant of 4.7 was used, as in our previous work.[5] Zero-point vibrational energy (ZPVE) and thermal (T = 298 K) vibrational corrections to the enthalpy were implemented. The values of the enthalpy difference between the aminal and enamine model compounds ($\Delta H = H_{aminal} - H_{enamine}$) were calculated as shown in Figure S12. The results show that enamine model compounds are more stable than the aminal ones for the three amines. From this series, the enthalpy of aminal model compound formed by DAP is only 6.69 kcal/mol higher from its enamine analogue, supporting that both compounds can be obtained experimentally. However, in the case of EDO and Jeff, the ΔH between the aminal and enamine model compounds are much higher, 12.51 and 13.12 kcal/mol, respectively. These larger ΔH values and the steric hindrance associated with the formation of larger cyclic compounds support the lack of aminal formation for EDO and Jeff.

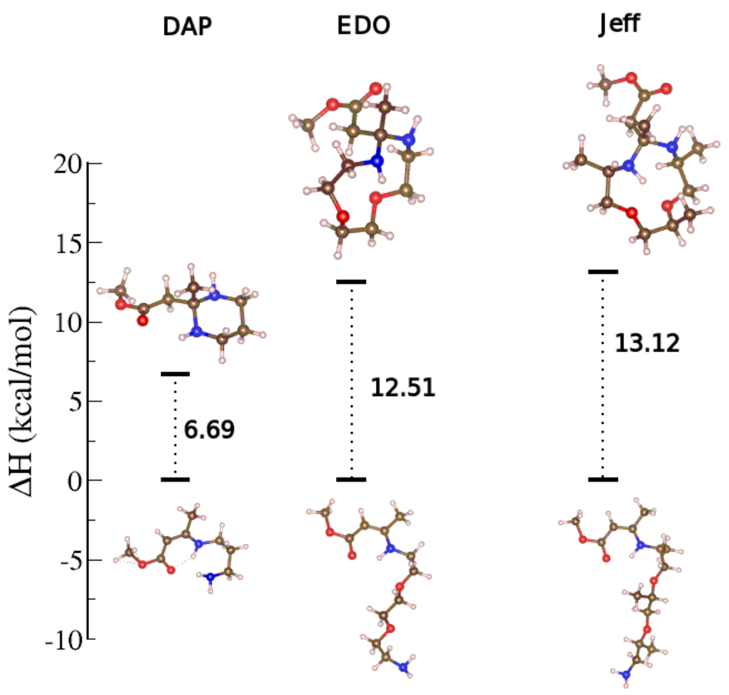

Figure S13. Enthalpy difference (ΔH, kcal/mol) between aminal and enamine model compounds formed by DAP, EDO and Jeff. Atoms are represented by the following color scheme: oxygen (red), nitrogen (blue), carbon (brown), and hydrogen (white).

References

(1) Frisch, M. J.; Trucks, G. W.; Schlegel, H. B.; Scuseria, G. E.; Robb, M. A.; Cheeseman, J. R.; Scalmani, G.; Barone, V.; Petersson, G. A.; Nakatsuji, H.; Li, X.; Caricato, M.; Marenich, A. V.; Bloino, J.; Janesko, B. G.; Gomperts, R.; Mennucci, B.; Hratchian, H. P.; Ortiz, J. V.; Izmaylov, A. F.; Sonnenberg, J. L.; Williams; Ding, F.; Lipparini, F.; Egidi, F.; Goings, J.; Peng, B.; Petrone, A.; Henderson, T.; Ranasinghe, D.; Zakrzewski, V. G.; Gao, J.; Rega, N.; Zheng, G.; Liang, W.; Hada, M.; Ehara, M.; Toyota, K.; Fukuda, R.; Hasegawa, J.; Ishida, M.; Nakajima, T.; Honda, Y.; Kitao, O.; Nakai, H.; Vreven, T.; Throssell, K.; Montgomery Jr., J. A.; Peralta, J. E.; Ogliaro, F.; Bearpark, M. J.; Heyd, J. J.; Brothers, E. N.; Kudin, K. N.; Staroverov, V. N.; Keith, T. A.; Kobayashi, R.; Normand, J.; Raghavachari, K.; Rendell, A. P.; Burant, J. C.; Iyengar, S. S.; Tomasi, J.; Cossi, M.; Millam, J. M.; Klene, M.; Adamo, C.; Cammi, R.; Ochterski, J. W.; Martin, R. L.; Morokuma, K.; Farkas, O.; Foresman, J. B.; Fox, D. J. *Gaussian 16 Rev. C.01*, Wallingford, CT, 2016.

(2) Weigend, F.; Ahlrichs, R., Balanced basis sets of split valence, triple zeta valence and quadruple zeta valence quality for H to Rn: Design and assessment of accuracy. *Phys. Chem. Chem. Phys.* **2005,** *7* (18), 3297-3305.

(3) Weigend, F., Accurate Coulomb-fitting basis sets for H to Rn. *Phys. Chem. Chem. Phys.* **2006,** *8* (9), 1057-1065.

(4) Tao, J.; Perdew, J. P.; Staroverov, V. N.; Scuseria, G. E., Climbing the Density Functional Ladder: Nonempirical Meta--Generalized Gradient Approximation Designed for Molecules and Solids. *Phys. Rev. Lett.* **2003,** *91* (14), 146401.

(5) Gómez Urreizti, E.; Gastearena, X.; Lam, A.; González de San Román, E.; Miranda, J. I.; Matxain, J. M.; Barroso-Bujans, F., Kinetics of heterogeneous polymerization of glycidol with B(C6F5)3 in toluene in the absence and presence of water. *Mater. Today Chem.* **2024,** *37*, 101993.
